# Setd4 controlled quiescent c-Kit^+^ cells contribute to cardiac neovascularization of capillaries beyond activation

**DOI:** 10.1038/s41598-021-91105-6

**Published:** 2021-06-02

**Authors:** Sheng Xing, Jin-Ze Tian, Shu-Hua Yang, Xue-Ting Huang, Yan-Fu Ding, Qian-Yun Lu, Jin-Shu Yang, Wei-Jun Yang

**Affiliations:** MOE Laboratory of Biosystem Homeostasis and Protection, College of Life, Sciences, Zhejiang University, Hangzhou, 310058 China

**Keywords:** Cell biology, Genetics, Molecular biology

## Abstract

Blood vessels in the adult mammal exist in a highly organized and stable state. In the ischemic heart, limited expansion capacity of the myocardial vascular bed cannot satisfy demands for oxygen supply and the myocardium eventually undergoes irreversible damage. The predominant contribution of endogenous c-Kit^+^ cells is understood to be in the development and homeostasis of cardiac endothelial cells, which suggests potential for their targeting in treatments for cardiac ischemic injury. Quiescent cells in other tissues are known to contribute to the long-term maintenance of a cell pool, preserve proliferation capacity and, upon activation, facilitate tissue homeostasis and regeneration in response to tissue injury. Here, we present evidence of a Setd4-expressing quiescent c-Kit^+^ cell population in the adult mouse heart originating from embryonic stages. Conditional knock-out of *Setd4* in *c-Kit-CreER*^*T2*^;*Setd4*^*f/f*^;*Rosa26*^*TdTomato*^ mice induced an increase in vascular endothelial cells of capillaries in both neonatal and adult mice. We show that Setd4 regulates quiescence of c-Kit^+^ cells by the PI3K-Akt-mTOR signaling pathway via H4K20me3 catalysis. In myocardial infarction injured mice, *Setd4* knock-out resulted in attenuated cardiomyocyte apoptosis, decreased infarction size and improved cardiac function. Lineage tracing in *Setd4-Cre*;*Rosa26*^*mT/mG*^ mice showed that Setd4^+^ cells contribute to each cardiac lineage. Overall, Setd4 epigenetically controls c-Kit^+^ cell quiescence in the adult heart by facilitating heterochromatin formation via H4K20me3. Beyond activation, endogenous quiescent c-Kit^+^ cells were able to improve cardiac function in myocardial infarction injured mice via the neovascularization of capillaries.

## Introduction

c-Kit, a type III receptor tyrosine kinase, is involved in multiple aspects of intracellular signaling. It participates in vital functions in many mammalian tissues including those of the heart. Lineage tracing showed that endogenous c-Kit^+^ cells have robust endothelial differentiation potential in their production of cardiac endothelial cells (ECs). However, they seem to have minimal myogenic commitment potential which ultimately proves inadequate to support efficient cardiac repair^1–3^. A recent study of this mechanism concluded that the observed functional benefits of cell therapy using exogenous c-Kit^+^ cells was probably due to acute inflammatory-based wound healing responses^4^. Exogenous use of bone marrow mononuclear cells has yielded similar conclusions. It has also been recently reported that endogenous c-Kit^+^ stem/progenitor cells (SPCs) participate in vascular repair in the aorta. Single-cell pseudotime analysis of scRNA-seq data suggests these cells undergo metabolic reprogramming during endothelial cell differentiation, in which AKT/mTOR-dependent glycolysis is critical for endothelial gene expression^5^. However, studies have so far failed to provide strong evidence for an exact mechanism of endogenous c-Kit^+^ cell action in the improvement of cardiac function beyond injury, such as in cases of myocardial infarction (MI).

Cellular quiescence is a conserved mechanism occurring in some somatic stem cells. These cells can be preserved in a quiescent state and then rapidly reactivated to proliferate and differentiate to replace cells lost in the process of normal homeostasis or to contribute to regeneration in response to tissue injury^6,7^. Epigenetic studies have shown that heterochromatin silences gene expression by virtue of its highly condensed structure and functions in this way in maintaining the reversibility of cellular quiescence^8–11^. Heterochromatin is highly involved in the trimethylation of lysine 20 of histone 4 (H4K20me3) and heterochromatin protein 1α (HP1α)^10–12^. In contrast, euchromatin is highly involved in the acetylation of lysine 9 of histone 3 (H3K9ac)^8,10,13^. More broadly, our models using diapause embryos of *Artemia*, human breast cancer stem cells (CSCs), and other contexts have their focus upon an evolutionarily conserved mechanism of cellular quiescence which is epigenetically regulated by facilitating heterochromatin formation with Set domain-containing protein 4 (Setd4) as a determinant^14,15^. In this study, we sought to identify whether Setd4-expressing quiescent c-Kit^+^ cells were present in the adult mouse heart. Upon confirmation, we continued to examine to what extent Setd4^+^ cells govern cardiac development in embryos and function in response to MI injury in adult mice.

Blood vessels in the heart arise from endothelial precursors during embryonic development. These are crucial for cardiac homeostasis by transporting oxygen and nutrients to the myocardium^16,17^. In adults, new coronary blood vessel formation, including coronary arteries and their branches of capillaries, are important clinical focus points in the development of treatments for cardiovascular diseases and in the potential for the promotion of cardiac regeneration^18,19^. However, in contrast to the active vessel growth in the embryo and the newborn, the adult myocardial coronary vascular bed rarely expands, except when provoked by stress or pathologic conditions. Even when it does so, its expansion capacity is limited and the heart can still incur injury due upon imbalance between oxygen supply and consumption in the ischemic myocardium^17^. Thus, angiogenesis derived from stem cells might be of particular value and have potential application in the promotion of therapeutic angiogenesis for patients with cardiac ischemia^16–18^. Mesenchymal stem cells (MSCs) and endothelial progenitor cells (EPCs) have emerged as potentially useful substrates for neovascularization, tissue repair and bioengineering^20–22^. MSCs are a rare population of fibroblast-like cells derived from the bone marrow stroma. They constitute approximately 0.001–0.01% of the nucleated cells in the marrow. EPCs are a heterogeneous group of endothelial cell precursors originating in the hematopoietic compartment of the bone marrow^22^. Investigators have often focused on early EPCs, also known as circulating proangiogenic cells (CACs) or PACs, because they can be easily and reproducibly isolated from the peripheral blood of healthy or diseased subjects^23–25^. These cells have been reported to prevent cardiomyocyte apoptosis and improve cardiac function via an increase in capillary number when injected into the injured heart^26^. However, the potential of endogenous c-Kit^+^ cells towards therapeutic angiogenesis for patients with cardiac ischemia remains unclear. Understanding how endogenous cells promote neovascularization in an injured region offers important clinical insight into developing treatments for cardiovascular diseases and in identifying potential contributors to cardiac regeneration.

In this study, we identify that Setd4 epigenetically regulates c-Kit^+^ cell quiescence by H4K20me3 catalysis via the PI3K-Akt-mTOR signaling pathway. We show that Setd4^+^ cells contribute to cardiac development and are substantially expressed in the c-Kit^+^ cells after birth. Beyond *Setd4* knock-out, quiescent endogenous c-Kit^+^ cells were activated and able to improve cardiac function in MI-injured mice via the neovascularization of capillaries. This occurred in a similar manner to the observation of c-Kit^+^ cells of previous studies, but now of endogenous rather than exogenous origin.

## Results

### Setd4 regulates the quiescence of c-Kit^+^ cells by facilitating heterochromatin formation

**Figure 1:**
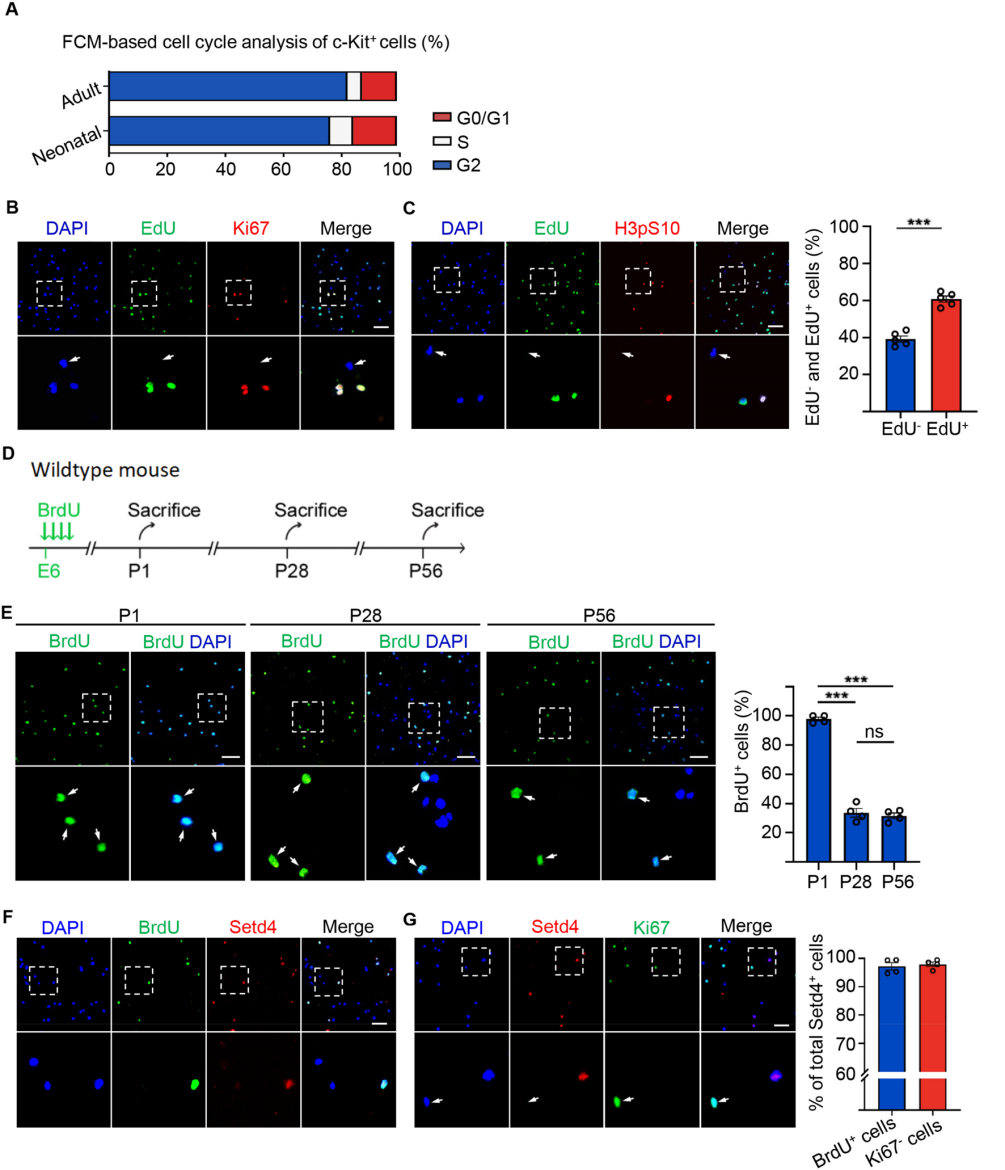
Identification of quiescent c-Kit^+^ cells. (**A**) FMC cell-cycle analysis of FACS-sorted c-Kit^+^ cells. (**B**, **C**) Representative immunofluorescence and quantification for c-Kit^+^ cells with EdU incorporation and proliferation marker, Ki67, H3pS10. n = 5 mice. (**D**) Experimental outline for BrdU retention assay. (**E**–**G**) Representative immunofluorescence and quantification for c-Kit^+^ cells with retained BrdU in P1, P28 and P56 hearts (**E**), Setd4 retained BrdU (**F**) and the cell proliferation marker, Ki67 in P56 hearts (**G**). n = 4 mice. Nuclei were stained with DAPI. Scale bars = 50 μm. All data are represented as mean ± SEM. Unpaired t test for (**B**) and (**F**), one-way ANOVA with Bonferroi’s correction for multiple comparisons test for (**E**). ***p < 0.001, *ns* not significant.

To identify quiescent c-Kit^+^ cells, we first isolated c-Kit^+^ cells from the hearts of neonatal and adult mice using fluorescence-activated cell sorting (Fig. S1A). Flow cytometry analysis of the c-Kit^+^ cell population showed that over 80% and 75% were in the G0/G1 phase in the adult and neonatal heart, respectively (Figs. 1A, S1B). In proliferating culture medium ^27^, we found that approximately 40% of sorted c-Kit^+^ cells failed to incorporate 5-ethynyl-2’-deoxyuridine (EdU) within two days and also lacked, Ki67 and the phosphorylation of H3S10 (H3pS10), as cell proliferation markers. This was in direct contrast to EdU incorporated c-Kit^+^ cells (Fig. 1B,C). Results strongly implied that these c-Kit^+^ cells were in a quiescent state. In addition, we serially administrated 5-bromo-2′deoxyuridine (BrdU) at embryonic day 6 (E6) into pregnant female mice (Fig. 1D). BrdU retention assays showed that BrdU was incorporated into almost all sorted c-Kit^+^ cells at one day after birth (P1) and approximately 30% of these cells had still retained BrdU when measurement was extended to 28 days (P28), or even 56 days (P56) after birth. This indicated that such cells had initially entered quiescence at the embryonic stage and that in such a quiescent state these cells had persisted into the adult heart (Fig. 1E).

**Figure 2:**
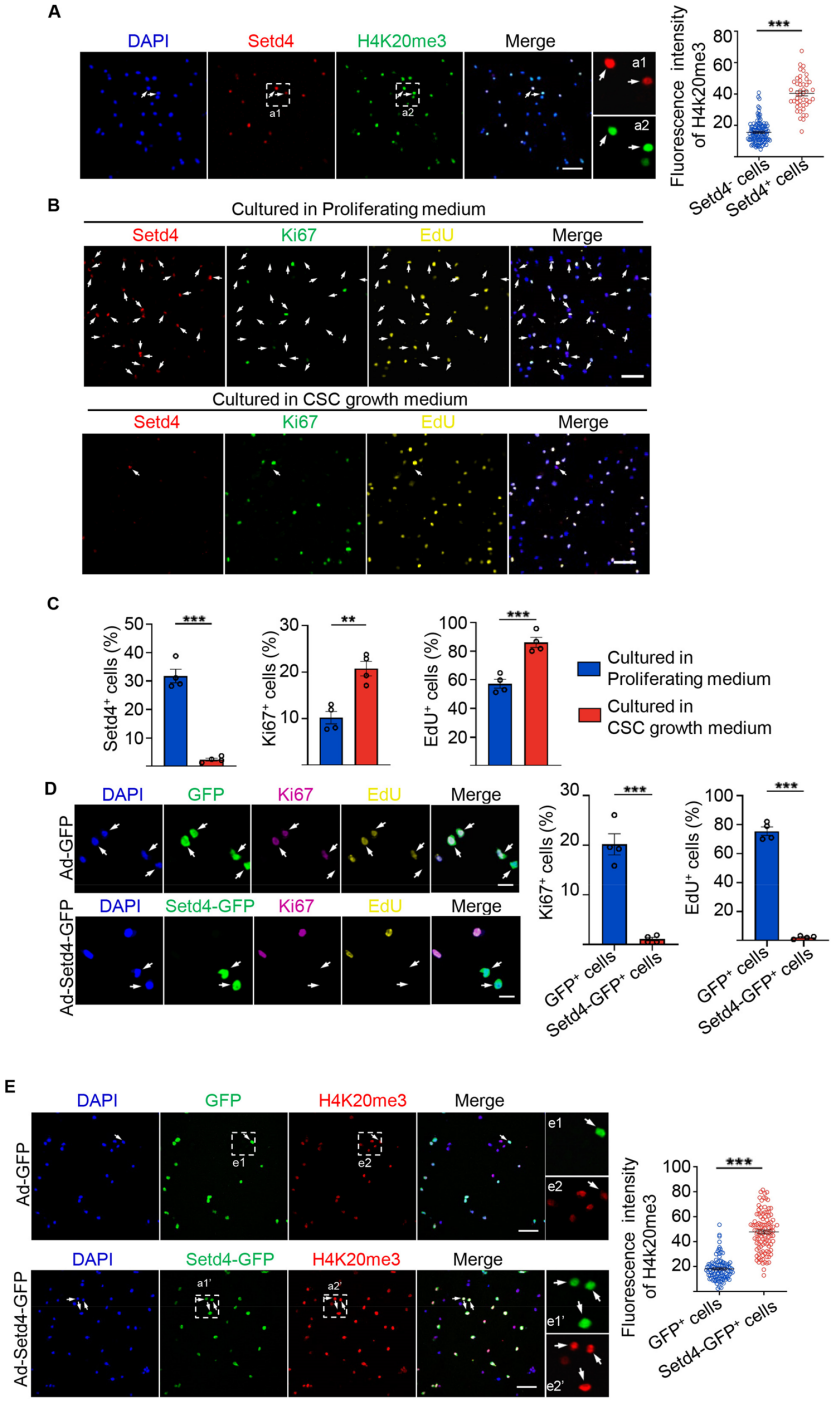
Setd4 regulates the quiescence of c-Kit^+^ cells by facilitating heterochromatin formation. (**A**) Identification and quantification for FACS-sorted c-Kit^+^ cells with high levels of Setd4 and H4K20me3. Scale bar = 50 μm. n = 173 cells from 3 mice. (**B**, **C**) Representative immunofluorescence and quantification for c-Kit^+^ cells cultured in proliferating medium or cardiac stem cell growth medium with Setd4 incorporation of EdU and Ki67. Scale bar = 50 μm. n = 4 mice. (**D**, **E**) Representative immunofluorescence and quantification for activated c-Kit^+^ cells affected by Ad-GFP and Ad-Setd4 with incorporation of EdU, Ki67 (**D**), n = 4 mice and H4K20me3 (**E**), n = 105 cells from 3 mice. Scale bars = 10 μm. Nuclei were stained with DAPI. All data are represented as mean ± SEM. Comparison of independent variables was conducted by two-tailed unpaired Student’s t test. **p < 0.01, ***p < 0.001.

Previously, we reported an evolutionarily conserved mechanism in which Setd4 controls cellular quiescence by facilitating heterochromatin formation via H4K20me3 catalysis^14,15^. In this, H4K20me3 localized to the promoter regions and regulated the expression of a set of genes in quiescent cells. Therefore, we sought to identify whether Setd4 also regulates c-Kit^+^ cell quiescence. As expected, a high level of Setd4 expression was observed in all BrdU retained quiescent c-Kit^+^ cells as compared to active c-Kit^+^ (BrdU^−^ c-Kit^+^) cells (Fig. 1F). In addition, Setd4-expressing (Setd4^+^) c-Kit^+^ cells lacked the expression of Ki67 and amplification of H3pS10 (Figs. 1G, S2A). These results suggest that quiescent c-Kit^+^ cells are indeed regulated by Setd4. Similarly, as markers for heterochromatin, high levels of H4K20me3 and HP1α were observed in Setd4^+^c-Kit^+^ cells, in contrast to those in Setd4 non-expressing (Setd4^−^) c-Kit^+^ cells (Figs. 2A, S2B). However, we did not observe any significant differences in levels of the euchromatin marker of H3K9ac between these cell types (Fig. S2C).

In addition, we found that after 2 days under in vitro cardiac stem cell culture medium conditions, the number of Setd4^+^c-Kit^+^ cells had significantly decreased and the expression of Ki67 and incorporation of EdU had significantly increased. This was in direct contrast to cells under proliferating culture medium conditions (Fig. 2B,C). These results indicate that quiescent c-Kit^+^ cells could be activated in vitro and that Setd4 is required for the maintenance of c-Kit^+^ cell quiescence. To validate the function of Setd4 in the regulation of c-Kit^+^ cell quiescence, a recombinant adenovirus expressing Setd4-IRES-GFP protein was transfected in activated c-Kit^+^ cells. Two days following *Setd4* overexpression, a significant increase of Setd4 expression was observed in c-Kit^+^ cells as detected by Western blot (Fig. S3A). In contrast to controls, we found that the expression of Ki67 in c-Kit^+^ cells was inhibited, and the incorporation of Edu failed after overexpression of *GFP-Setd4*, (Fig. 2D). Notably, we observed a significant increase of H4K20me3 and HP1α in *GFP-Setd4* overexpressed c-Kit^+^ cells, in contrast to controls (Figs. 2E, S3B). However, the level of H3K9ac showed no significant difference between *GFP-Setd4* and *GFP* overexpressed c-Kit^+^ cells (Fig. S3C). Taken together, Setd4 regulates c-Kit^+^ cells quiescence by facilitating heterochromatin formation via H4K20me3 catalysis.

### Setd4^+^ cells contribute to cardiac lineages in embryos

**Figure 3:**
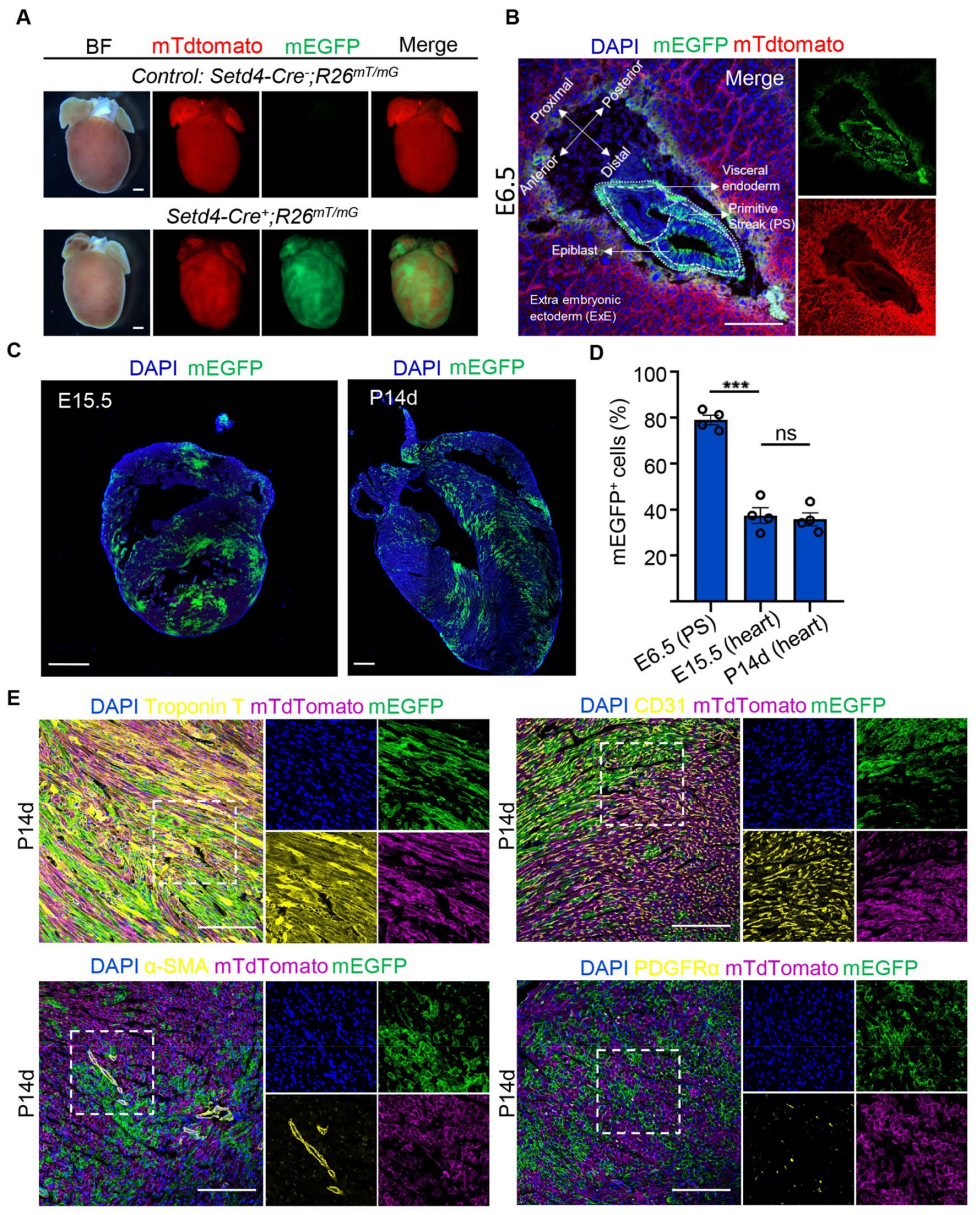
Setd4^+^ cells contribute to cardiac development in embryos. (**A**), Fluorescent images of hearts of *Setd4-Cre*^+^*;Rosa26*^*mT/mG*^ mice and the control *Setd4-Cre*^*−*^*;Rosa26*^*mT/mG*^ mice. Scale bars = 500 μm. (**B**, **C**) Representative immunofluorescence for recombinant mG^+^ cells in E6.5 germ layers (**B**), E15.5 and P14 hearts (**C**). PS: primitive streak; ExE: extra embryonic ectoderm. Scale bars = 500 μm. (**D**) Quantification for recombinant mG^+^ cells in E6.5 PS, E15.5 and P14 hearts. (**E**) Representative immunofluorescence for recombinant mG^+^ cells with cardiomyocyte, EC, smooth muscle cell and fibroblast markers, Troponin T, CD31, α-SMA and PDGFRα, respectively, in the P14 heart. Scale bars = 50 μm. Nuclei were stained with DAPI. All data are represented as mean ± SEM. n = 4 mice. One-way ANOVA with Bonferroi’s correction for multiple comparisons test for (**D**). ***p < 0.001, ns: not significant.

Heart development is one of the earliest events of vertebrate organogenesis^28,29^. Cardiac progenitor cells (CPCs) give rise to the myocardium, endocardium, epicardium, smooth muscle, fibroblasts, and endothelium of coronary vessels in the mammalian heart. In the mouse embryo, CPCs originate between embryonic day (E) 6.25 and E7.5 from nascent mesoderm cells in the primitive streak (PS)^30–32^. In this study, we generated *Setd4-Cre*;*Rosa26*^*mT/mG*^ mice (Fig. S4A) and used *Setd4-Cre*^*−*^*;Rosa26*^*mT/mG*^ mice line for controls. We noticed significant fluorescence in the postnatal *Setd4-Cre*^+^*;Rosa26*^*mT/mG*^ mouse heart, which indicated that Setd4 might contribute to heart development (Fig. 3A). To clarify this, we performed lineage tracing of Setd4^+^ cells in the hearts of E6.5, E15.5 and postnatal day (P) 14 mice and found that mEGFP recombinant (mG^+^) cells had distributed to over 75% cells in the PS by E6.5 (Fig. 3B). After mouse gastrulation, CPCs in the PS finally give rise to the mouse heart. Lineage tracing results showed that mG^+^ cells comprised approximately 37.4% cells in the E15 mouse heart and was maintained at this level beyond birth (P14) (Fig. 3C,D). This indicated that Setd4^+^ cells that originated from early embryonic stages then contributed to early cardiac development. In addition, mG^+^ cells were also observed to contribute to all cardiac lineages of cardiomyocytes, ECs, smooth muscle cells and fibroblasts by detecting their respective markers (Troponin T, CD31, α-SMA and PDGFRα, respectively) in E15 and P14 hearts (Figs. 3E, S5A). However, using immunofluorescence analysis we found that after birth Setd4 protein was substantially expressed in the c-Kit^+^ cells, but only minimally in c-Kit^-^ cells (P1) (Fig. S5B). These results indicate that Setd4^+^ cells originate from early embryonic stages, contribute to almost all cardiac lineages in embryos and persist after birth. Setd4 is specifically expressed in c-Kit^+^ cells where Setd4 may epigenetically control c-Kit^+^ cell quiescence.

### Conditional knock-out of *Setd4* induced the generation of newborn ECs in neonatal and adult mice

**Figure 4:**
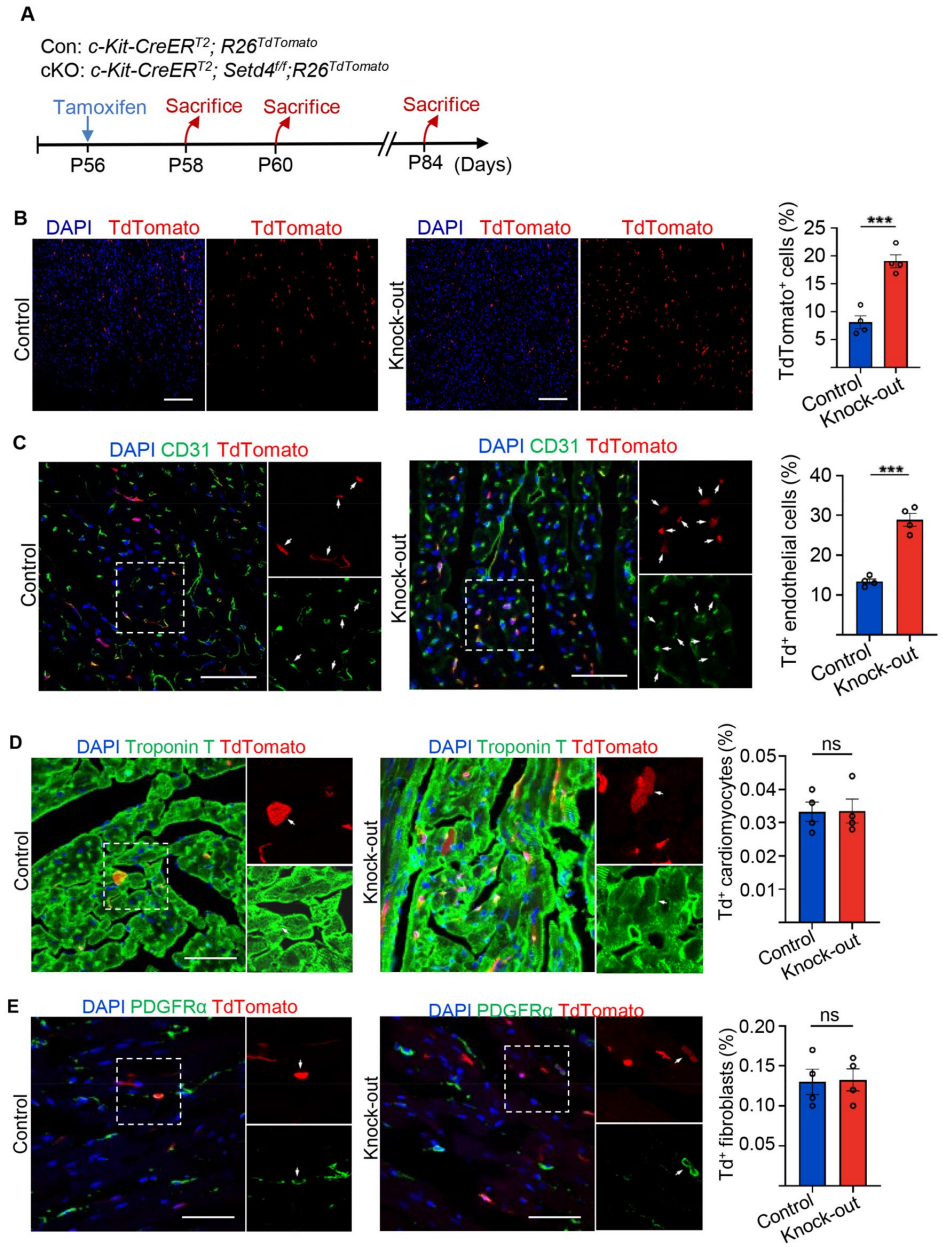
Knock-out of *Setd4* induced generation of newborn ECs in adult mice. (**A**) Experimental outline for lineage tracing of adult mice after knock-out of *Setd4*. (**B**) Representative immunofluorescence and quantification for recombinant cells after four weeks of *Setd4* knock-out. Scale bars = 200 μm. (**C–E**) Identification and quantification for c-Kit^+^ cell-derived ECs (Td^+^CD31^+^) (**C**), cardiomyocytes (Td^+^Troponin T^+^) (**D**) and fibroblasts (Td^+^PDGFRα^+^) (**E**) after four weeks of *Setd4* knock-out. Scale bars = 50 μm. Arrows indicate recombined cells and positive staining. Nuclei were stained with DAPI. All data are represented as mean ± SEM. n = 4 mice. Comparison of independent variables was conducted by two-tailed unpaired Student’s t test. ***p < 0.001, ns: not significant.

The functions of quiescent cells could only be examined after their activation. To activate Setd4^+^c-Kit^+^ cells in adults, we generated conditional *Setd4* knock-out (*c-Kit-CreER*^*T2*^;*Setd4*^*f/f*^;*Rosa26*^*TdTomato*^) contrasted to control (*c-Kit-CreER*^*T2*^;*Rosa26*^*TdTomato*^) mice (Fig. S4B). After 48 h post-induction of Tamoxifen (TAM) (Fig. 4A), Western blot analysis confirmed that the expression of Setd4 in sorted Td^+^ cells had been almost entirely abolished. This indicated that *Setd4* knock-out was successful (Fig. S6A,B). After 4 days of TAM-induction in mice, results detecting the proliferation marker Ki67 in Td^+^ cells showed significantly increased proliferation levels in *Setd4* knock-out mice in contrast to control mice (Fig. S6C). After 4 weeks of TAM-induction in mice, Td^+^ cells had increased approximately 1.4-fold in *Setd4* knock-out mice (*c-Kit-CreER*^*T2*^;*Setd4*^*f/f*^;*Rosa26*^*TdTomato*^) compared to control mice (Fig. 4B). This indicated that the knock-out of *Setd4* had led to the activation of quiescent c-Kit^+^ cells and a corresponding increase of their progenies in adults.

To identify the progenies beyond *Setd4* knock-out in adult *c-Kit-CreER*^*T2*^;*Setd4*^*f/f*^;*Rosa26*^*TdTomato*^ mice, the expression of CD31, Troponin T and PDGFRα was detected. Results showed a significant increase in the number of ECs (Td^+^CD31^+^), but no changes in the numbers of cardiomyocytes and fibroblasts in *Setd4* knock-out mice, in contrast to control mice (Fig. 4C–E). This was validated by the alternative detection of VE-cadherin, Desmin and Sox9, markers of ECs, cardiomyocytes and fibroblasts, respectively (Fig. S7A–C). In addition, we performed *Setd4* knock-out in neonatal mice. After injection of TAM at postnatal day one (P1), the mice were sacrificed 4 weeks later and processed for analysis (Fig. S8A). As observed in the adults, the number of Td^+^ cells had increased after *Setd4* knock-out in which the majority had adopted an ECs fate (Td^+^CD31^+^), but had also minimally contributed to cardiomyocytes (Td^+^Troponin T^+^) and fibroblasts (Td^+^PDGFRα^+^) (Fig. S8B–E). Taken together, our results demonstrate that the deletion of *Setd4* promotes regeneration of ECs in both neonatal and adult mice.

### Knock-out of *Setd4* contributes to neovascularization of capillaries in the heart

**Figure 5:**
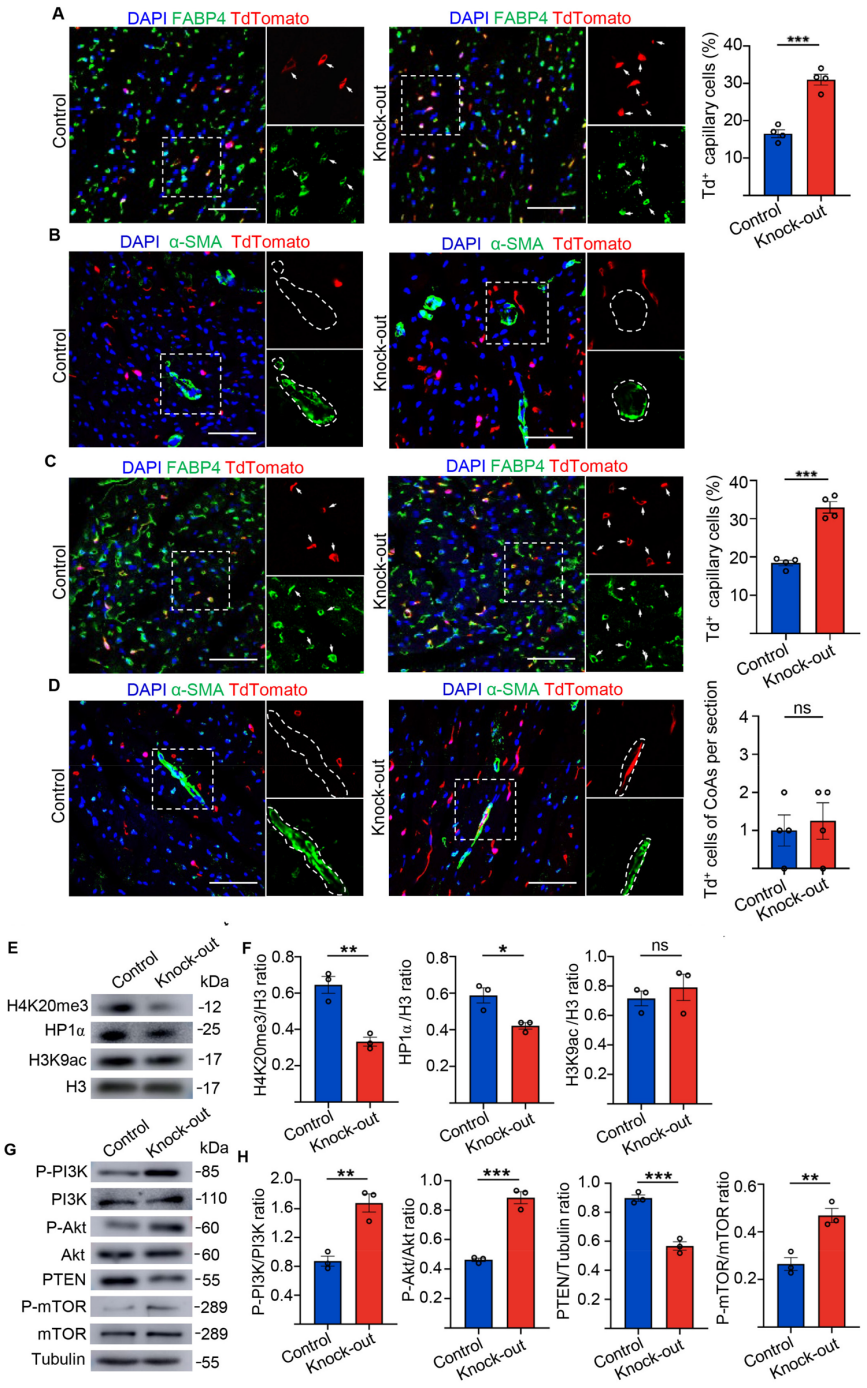
Knock-out of *Setd4* contributes to neovascularization of capillaries in adult heart. (**A**) Representative immunofluorescence and quantification for recombinant cells with coronary endothelial cell marker FABP4 in adult mice after four weeks of *Setd4* knock-out. n = 4 mice. (**B**) Immunostaining for Td and smooth muscle cell marker of α-SMA shows no Td^+^ coronary arteries generated in adult mice after four weeks of *Setd4* knock-out. n = 4 mice. (**C**, **D**) Immunostaining for Td and FABP4 (**C**) and α-SMA (**D**) and quantification for recombinant cells of capillary and coronary artery potential in neonatal mice after four weeks of *Setd4* knock-out. n = 4 mice. Scale bars = 50 μm. Nuclei were stained with DAPI. (**E**, **F**) Representative western blot analysis (**E**) and relative band densitometric analysis (**F**) of H4K20me3, HP1α and H3K9ac. n = 3. (**G**) Representative western blot analysis of PI3K, Akt, mTOR with their phosphorylated forms and PTEN. (**H**) The relative band densitometric analysis of P-PI3K, P-Akt, P-mTOR and PTEN. n = 3. All data are represented as mean ± SEM. n = 4 mice. Comparison of independent variables was conducted by two-tailed unpaired Student’s t test. ***p < 0.001.

ECs play a critical role in blood vessel formation, both during early development and in the adult heart. During embryonic development, blood vessels arise from endothelial precursors which share an origin with haematopoietic progenitors^16,17^. It has been reported that the injection of endothelial progenitor cells into adult mice can preserve cardiac function by neovascularization in response to myocardial ischemia^33^. Therefore, we aimed to identify whether these newborn ECs, as produced by Setd4^+^c-Kit^+^ cells, could contribute to blood vessel formation in both neonatal and adult mice. After 4 weeks of *Setd4* knock-out in adult mice (P56), we found that the newborn ECs produced by Setd4-expressing c-Kit^+^ (Td^+^) cells expressing the capillary marker FABP4, had significantly increased in contrast to those in the control mice (Fig. 5A). However, we did not observe Td^+^ cells inner of coronary arteries as marked by α-SMA, a marker of smooth muscle cells (Fig. 5B). These results indicated that these newborn ECs had been able to form capillaries but not coronary arteries. Similarly, in neonatal mice (P1), most newborn ECs (Td^+^) adopted a capillary fate (Td^+^FABP4^+^), but also minimally contributed to coronary arteries, as marked by α-SMA after *Setd4* knock-out, in contrast to controls (Fig. 5C,D). These results indicated that quiescent c-Kit^+^ cells were able to produce ECs and contribute to the generation of capillaries for cardiac homeostasis. In addition, deletion of *Setd4* in quiescent c-Kit^+^ cells clearly promoted neovascularization of capillaries, but also contributed, albeit infrequently, to coronary arteries in neonatal mice.

We then investigated the mechanisms of c-Kit^+^ cell quiescence regulation. Here, we sorted Td^+^ cells from *Setd4* knock-out and control mice after 48 h post-induction of TAM in adult hearts. Western blot analysis showed that *Setd4* knock-out induced significant decreases in H4K20me3 and HP1α expression but resulted in no significant alteration in H3K9ac in c-Kit cells (Fig. 5E,F). In addition, we found that the levels of PI3K, Akt, and mTOR phosphorylation were significantly increased, but that PTEN expression was decreased after *Setd4* knock-out (Fig. 5G,H). Thus, we concluded that Setd4 controls c-Kit^+^ cell quiescence by the PI3K-Akt-mTOR signaling pathway via H4K20me3 catalysis.

### Knock-out of *Setd4* preserves cardiac function beyond MI-induced injury

**Figure 6:**
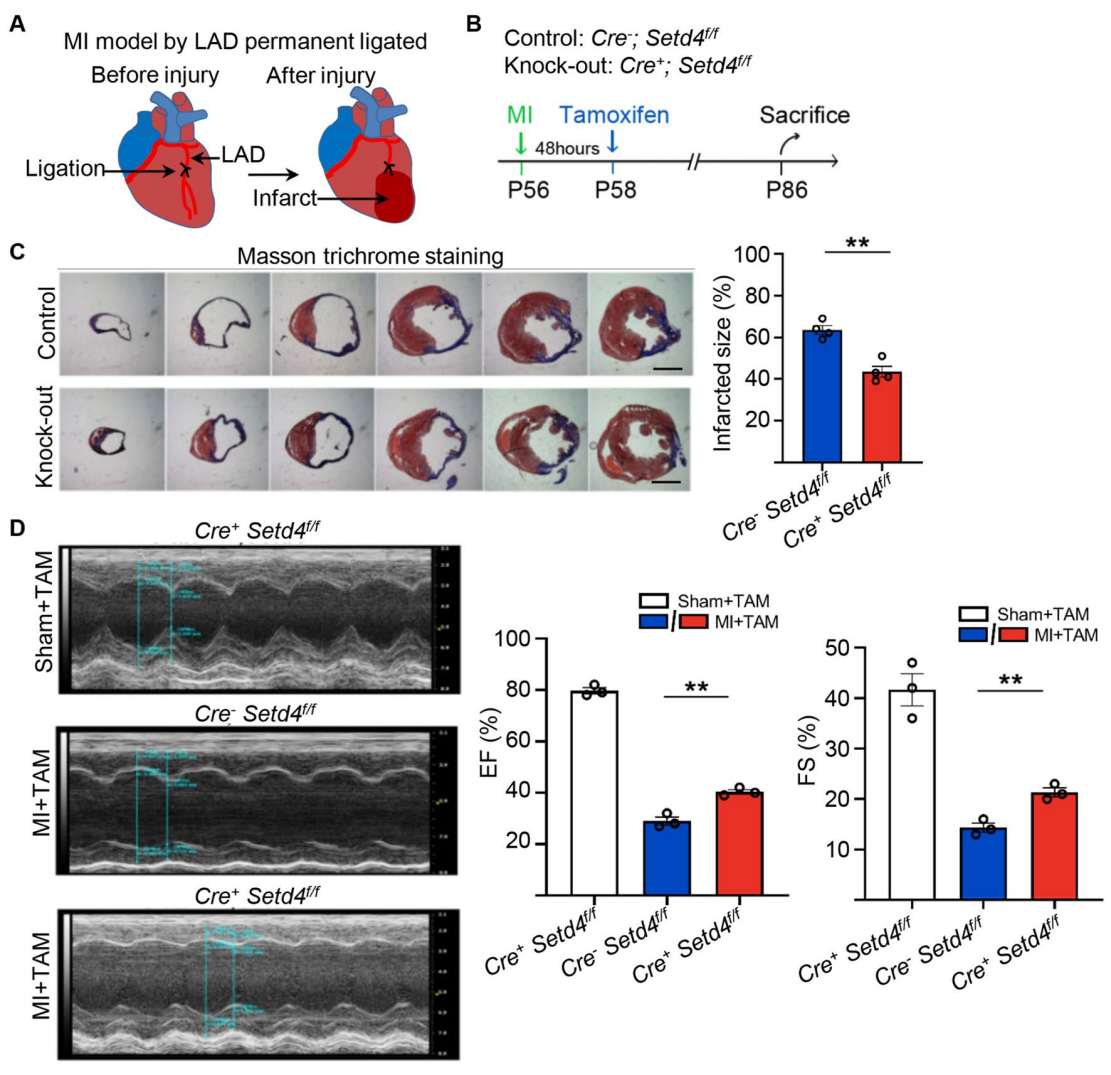
Knock-out of *Setd4* improves cardiac function in MI-injured mice. (**A**) Diagram of MI-induced injury. (**B**) Experimental outline for injured hearts of *c-Kit-CreER*^*T2*^*(Cre*^+^*)*;*Setd4*^*f/f*^ and *c-Kit-CreER*^*T2−*^*(Cre*^*-*^*)*;*Setd4*^f^^*/f*^ mice. (**C**) Masson trichrome staining and quantification for infarction size in hearts of *Setd4* knock-out and control mice. Scale bars = 2 mm. n = 4 mice. (**D**) Detection of left ventricular function by transthoracic 2-dimensional echocardiography of *Setd4* knock-out and control mice. n = 3 mice. All data are represented as mean ± SEM. Unpaired t test for (**C**), two-way ANOVA with Bonferroi’s correction for multiple comparisons test for (**D**). **p < 0.01.

Any potential factor that may relate to vascular regeneration in the adult heart would require consideration for its therapeutic value. It has already been demonstrated that transplanted cells can contribute to the formation of functional vessels, restoring blood flow to ischemic regions for improved cardiac function^26,33,34^. As we have shown, quiescent c-Kit^+^ cells have the capacity to generate more capillaries upon the deletion of *Setd4*. We therefore investigated whether the deletion of *Setd4* in quiescent c-Kit^+^ cells would lead to improved cardiac function beyond injury. We performed TAM-induction after 2 days of MI injury caused by left anterior descending permanent ligation in *Setd4* knock-out mice (*c-Kit-CreER*^*T2*^;*Setd4*^f^^*/f*^) and control mice (*c-Kit-CreER*^*T2−*^;*Setd4*^f^^*/f*^) (Fig. 6A,B). Masson-trichrome stain analysis showed that there was no significant difference of infarct size between the two groups within one week after tamoxifen induction. Over time, myocardial ischemia induced by MI presented obvious myocardial fibrosis in both groups. And the infarct size was significantly lower in the *Setd4* knock-out mice than in control group after 4 weeks of tamoxifen induction (Figs. 6C and S12). In addition, to examine the left ventricular function, transthoracic 2-dimensional echocardiography was performed. The ejection fraction and fractional shortening were 79.7% and 41.7% in the group of sham mice, but 29.0% and 14.3% in the group of control mice after MI injury, respectively. Interestingly, these values significantly increased to 40.3% and 21.3% after *Setd4* knock-out in *c-Kit-CreER*^*T2*^;*Setd4*^*f/f*^ mice (Fig. 6D). This indicated that deletion of *Setd4* was able to preserve cardiac function in response to MI-induced injury.

**Figure 7:**
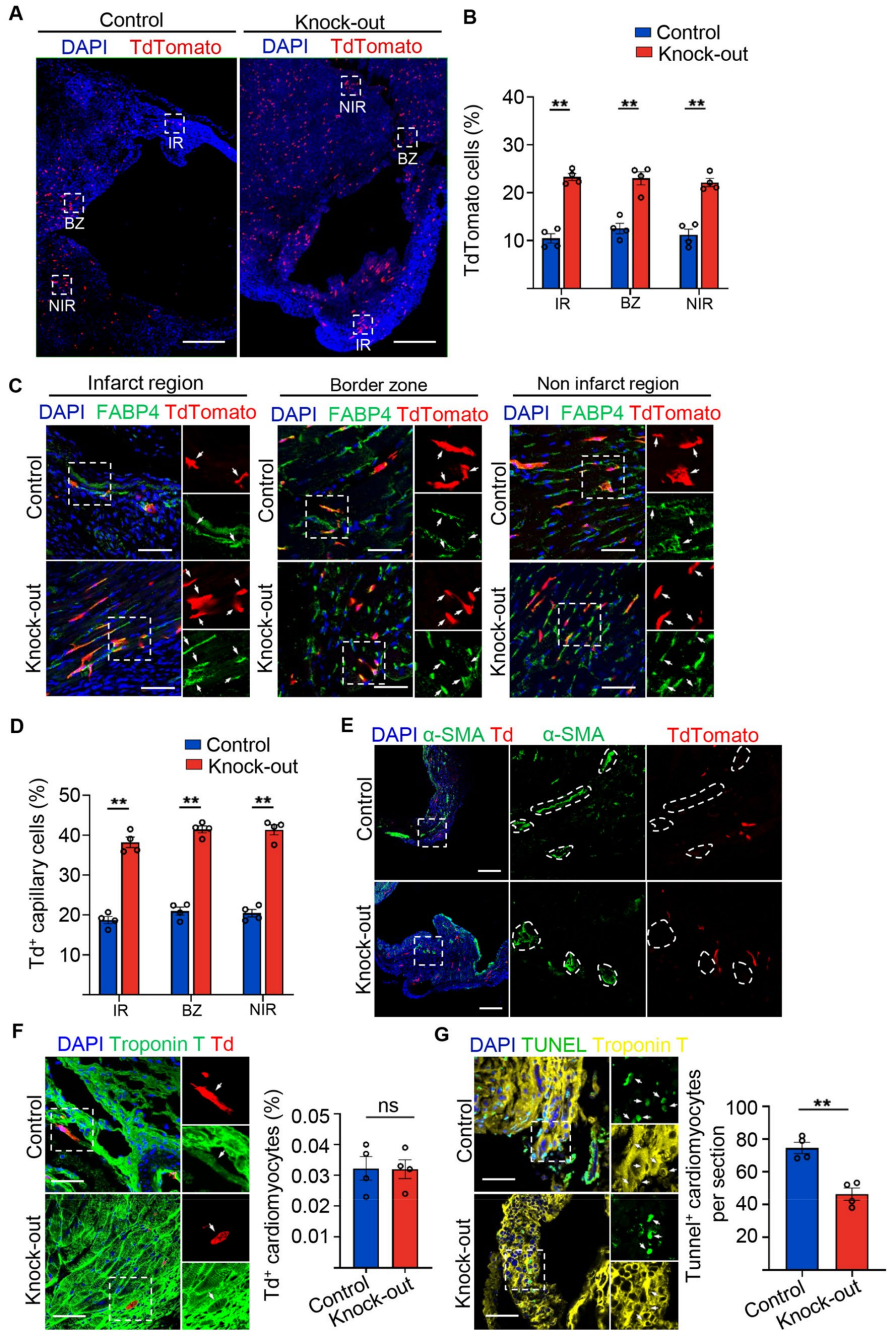
Knock-out of *Setd4* inhibits cardiomyocyte apoptosis in MI-injured mice. (**A**, **B**) Representative immunofluorescence (**A**) and quantification (**B**) for recombinant cells. Scale bars = 500 μm. In the three areas of the MI-injured left ventricle after four weeks of *Setd4* knock-out. Scale bars = 500 μm. (**C**, **D**) Immunostaining (**C**) and quantification (**D**) for recombinant cells of capillary (Td^+^FABP4^+^) in the three areas of the MI-injured left ventricle. Scale bars = 50 μm. (**E**, **F**) Immunostaining for α-SMA (Scale bars = 200 μm) (**E**) and Troponin T (Scale bars = 50 μm) (**F**) after four weeks of *Setd4* knock-out. (**G**) Representative immunofluorescence and quantification for apoptotic cardiomyocytes by performing TUNEL assay after two weeks of *Setd4* knock-out. Scale bars = 50 μm. Nuclei were stained with DAPI. All data are represented as mean ± SEM. n = 4 mice. Unpaired t test for (**F**, **G**), two-way ANOVA with Bonferroi’s correction for multiple comparisons test for (**B**, **D**). **p < 0.01. *ns* not significant, *IR* infarction region, *BZ* border zone, *NIR* non-infarction region.

After *Setd4* knock-out, we found that the number of Td^+^ labelled cells in all regions of the infarction region (IR), border zone (BZ) and non-infarction region (NIR) had significantly increased in contrast to controls (Fig. 7A,B). By detecting the corresponding markers of FABP4 and Td signals inner of coronary arteries marked by α-SMA, we were able to establish that these progenies of quiescent c-Kit^+^ (Td^+^) cells contributed to ECs that adopted capillary fates but made minimal contributions to those adopting to coronary arteries fates (Fig. 7C–E). They also rarely contributed to cardiomyocytes in *Setd4* knock-out or control mice (Fig. 7F). To examine whether the newly generated capillaries could inhibit the apoptosis of cardiomyocytes in response to injury, a terminal deoxynucleotidyl transferase-mediated dUTP nick-end labeling (TUNEL) assay was performed after *Setd4* knock-out in MI mice. It was observed that apoptosis in cardiomyocytes was inhibited by *Setd4* knock-out, in contrast to controls (Fig. 7G). We concluded that the activation of quiescent c-Kit^+^ cells by *Setd4* deletion promotes the neovascularization of capillaries, inhibits cardiomyocyte apoptosis and thus preserves cardiac function in response to MI-induced injury in the adult heart.

## Discussion

In this study, lineage tracing revealed that Setd4^+^ cells had contributed to over 75% cells in the PS at E6.5 and contributed to approximately 37.4% cells of cardiac lineages including cardiomyocytes, ECs, smooth muscle cells and fibroblasts in the heart. Thus, Setd4^+^ cells may act as multipotent cardiovascular progenitors to govern cardiac development in embryos. In the adult heart, Setd4 was specifically expressed in c-Kit^+^ cells where Setd4 epigenetically controls c-Kit^+^ cell quiescence. These quiescent c-Kit^+^ cells may represent a developmental remnant that remain in a quiescent state via epigenetic regulation by Setd4, and then persist into the adult heart. Quiescent c-Kit^+^ cells are then able to reactivate and maintain the function of neovascularization when the requirement exists for replenishment of any c-Kit^+^ cells that have been depleted, whether gradually and over extended periods, as in normal homeostasis, or more abruptly due to MI-induced injury.

Several recent studies have shown the profound effect of inflammatory response determining the outcome of myocardial injuries. The clear distinction between the inflammatory response and the activation of quiescent cells needs to be acknowledged here, especially when both processes target the same ends. Indeed, we did notice several recent studies of inflammatory response effectively influencing the outcome of myocardial injuries. It is clear that the majority of these inflammatory response related reports used exogenous injectants to cause immune reactions and their results clarified cell therapies well. However, our results differed in that in this study we clearly highlighted the effect of quiescent c-Kit^+^ cells derived from the heart in response to injury after Setd4 KO and we did not observe any immune response when Setd4 was knocked out in quiescent c-Kit^+^ cells.

C-Kit^+^ cells, MSCs and EPCs have all been shown to act to improve cardiac function after myocardial infarction. However, whether one cell type is superior has long been an area of speculation with few clinical head-to head trials attempted. According to recent reports, MSCs display a greater therapeutic potential due to their multipotent differentiation ability toward vascular smooth muscle cells, endothelial cells and other cells types^21^. EPCs including CACs and PACs, have been shown to have direct angiogenic actions and/or to be able to support angiogenesis. However, they appear inferior to MSCs that possess a broader differentiation potential that can also include the pericytes or smooth muscle cells that would be required for mural cell stabilization of vessels^17^. Our results showed endogenous quiescent c-Kit^+^ cells could be activated to promote the generation of endothelial cells, but also minimally contribute to smooth muscle cells. We speculate that exogenous injectants like MSCs and EPCs would mobilize and activate endogenous quiescent c-Kit^+^ cells, and this synergy could effectively promote angiogenesis, inhibit apoptosis and modulate immunoreaction in response to cardiac ischemic injury.

Previously, we showed that Setd4 epigenetically controls breast CSC quiescence by facilitating heterochromatin formation via H4K20me3 catalysis, in which H4K20me3 localizes to the promoter regions and regulates the expression of a set of genes in quiescent CSCs^15^. This pattern of gene expression has also been identified by bulk and single quiescent breast CSC RNA sequencing analysis. Similar processes are observed in this study where quiescent c-Kit^+^ cells express abundant Setd4 and H4K20me3. We thus propose that c-Kit^+^ cell quiescence in the heart is regulated by Setd4 via an evolutionarily conserved mechanism. In addition, we found that the PI3K-Akt-mTOR signaling pathway was involved in the activation process of quiescent c-Kit^+^ cells after *Setd4* knock-out and show that Setd4 epigenetically regulates c-Kit^+^ cell quiescence by the PI3K-Akt-mTOR signaling pathway via H4K20me3 catalysis.

After acute MI, infarcts affecting 40% or more of left ventricle often lead to severe myocardial dysfunction that may result in sudden cardiac death or acute congestive heart failure^35^. Although neoangiogenesis within the infarcted tissue appears to be an integral component of the remodeling process, the capillary network cannot keep pace with tissue growth and is unable to support the greater demands of the injured myocardium^36–39^. In the current study, a significant mitigation of MI was observed after *Setd4* knock-out in which neovascularization of capillaries significantly increased. This indicates that neovascularization of capillaries derived from endogenous quiescent c-Kit^+^ cells contributes to the repair of MI-induced injury. We found that apoptosis in cardiomyocytes was inhibited by *Setd4* knock-out, in contrast to controls (Fig. 7G). We propose that this neovascularization holds benefits for cardiomyocytes in which increased capillaries generated from quiescent c-Kit^+^ cells meet the demands of anoxic cardiomyocytes and mitigate MI-induced injury by inhibiting cardiomyocyte apoptosis.

In the cardiac regeneration–stem cell debate, some have argued vociferously against the idea that the heart may contain its own cardiomyocyte-producing stem cell, often citing the failure of new-cardiomyocyte generation in rodent models^1–3^. For some, this seems to have led to a more general scepticism relating to endogenous sources of cardiac regeneration. Conversely, exogenous pluripotent stem cells have been confirmed to serve as mediators of cardiac regeneration of infarcted tissue in cardiovascular medicine^26,33,34^. Previous reports have shown that the injection of c-Kit^+^ cells to an injured area is able to improve cardiac function by robust de novo myogenesis derived directly from the injected cells^40–42^. Repeated studies by independent groups confirmed functional improvements but failed to observe the occurrence of new cardiomyocyte formation derived from such injected cells^43,44^. Therefore, the exact mechanism of how c-Kit^+^ cells act in cardiac improvement has remained unknown.

Despite this current lack of understanding of the exact mechanisms, implantation of bone marrow mononuclear cells into MI-injured mice have also been cited to improve cardiac function. In these, significantly increased number of capillaries were seen to occur in a similar manner as we observed with our model of *Setd4* knock-out in c-Kit^+^ cells. Our model of *Setd4* knock-out in c-Kit^+^ cells now confirm similar aspects of cardiac regeneration, improved cardiac function and capillary increase, from an endogenous source in the heart. Therefore, endogenous quiescent c-Kit^+^ cells, upon their activation, may hold great therapeutic potential towards vascular regeneration in response to ischemic injury.

## Conclusion

In conclusion, this study demonstrates that Setd4 epigenetically regulates the quiescence of c-Kit^+^ cells by H4K20me3 via the PI3K-Akt-mTOR signaling pathway. Activation of endogenous quiescent c-Kit^+^ cells by conditional knock-out of the *Setd4* gene in c-Kit^+^ cells induced an increase in vascular endothelial cells of capillaries during homeostasis and in response to the ischemic injury, which led to efficient mitigation of myocardial infarction. Setd4^+^ cells were also demonstrated to have multipotential differentiation for cardiovascular lineages in the embryonic heart. Our findings suggest Setd4 and/or Setd4^+^ quiescent c-Kit^+^ cells may be also used as key targets in clinical treatment for wide range of cardiac diseases.

## Methods

### Mice

All mice experiments were approved by and performed following the guidelines of the Institutional Animal Care and Use Committee at Zhejiang University and the study was carried out in compliance with the ARRIVE guidelines. The *Setd4-Cre* line was generated by Shanghai Model Organisms Center, Inc. using standard methods of CRISPR/Cas9. The offspring of *Setd4-Cre* were mated with *Rosa26-TdTomato* transgenic mice (no.007614; from The Jackson Laboratory) as previously reported^45^. Mice with a pair of loxP sites flanking exon6 of *Setd4* were generated by Shanghai Model Organisms Center, Inc, then, they were mated with FLP mice to excise the Neo cassette. The offspring were mated with *c-Kit-CreER*^*T2*^ transgenic mice (purchased from Shanghai Model Organisms Center, Inc.), and *Rosa26-TdTomato* transgenic mice. All experimental mice were maintained on a C57BL/6; 129 background. Genotyping was performed by conventional PCR on genomic DNA isolated from mouse-tails using standard procedures according to the instruction of Quick Genotyping Assay Kit for Mouse Tail (Beyotime, China D7283S).

### Tamoxifen administration

Tamoxifen (20 mg/ml, Sigma, T5648) was prepared in a 9:1 corn oil (Sigma) to ethanol mixture and administered by intraperitoneal injection at the indicated time points (0.1–0.15 mg tamoxifen/g mouse body weight) as previously reported^46^.

### Myocardial infarction

Adult mice (8–9 weeks old) were anaesthetized with 0.05 mg/g pentobarbital sodium and placed in a supine position on a heating pad (37 °C). Animals were ventilated using a MiniVent Type 845 mouse ventilator (stroke volume, 250 ml; respiratory rate, 120 breaths per minute). Myocardial infarction (MI) was achieved by permanent ligation of the left anterior descending coronary artery with a 6–0 prolene suture. Then, thoracic wall incisions were sutured with 6–0 non-absorbable silk sutures. A absorbable suture material was used to suture the muscle layer and epidermis as previously reported^2^. Sham-operated animals served as surgical controls and were subjected to the same procedures as the experimental animals with the exception that the left anterior descending coronary artery was not ligated. Immediately following surgery mice received sub-cutaneous injections of buprenorphine (0.05–0.1 mg/kg) for analgesia once a day for a total of three days.

### Echocardiographic analysis

Echocardiography was performed blindly using the Vevo 770 High-Resolution Micro-Imaging System (Visual Sonics) with a 15 MHz linear-array ultrasound transducer. The left ventricle was assessed in both parasternal long-axis and short-axis views at a frame rate of 120 Hz. End-systole or end-diastole were defined as the phases in which the left ventricle appeared the smallest and largest, respectively, and used for ejection-fraction measurements. To calculate the shortening fraction, left-ventricular end-systolic and end-diastolic diameters were measured from the left-ventricular M-mode tracing with a sweep speed of 50 mm/s at the papillary muscle as previously reported^47^. Imaging and calculations were done by an individual who was blinded to the treatment applied to each animal and this code was broken only after all data was acquired. At the end of the experiments, Animals were sacrificed by barbiturate overdose (150 mg/kg pentobarbital sodium, intraperitoneal injection) and cervical dislocation, and their hearts harvested for histological studies.

### TUNEL assay

To detect apoptotic cardiomyocytes, injured hearts were fixed in 4% paraformaldehyde (pH 8.0), dehydrated and embedded in optimum cutting temperature (O.C.T., Sakura) and frozen at − 80 °C. Terminal deoxynucleotidyl transferase-mediated dUTP nick-end labeling (TUNEL) assay was then performed on fresh frozen sections (10 μm thickness each). ApopTag® Plus in Situ Apoptosis Fluorescein Detection Kit (Cat #S7111 Millipore) was used according to the manufacturer’s procedure. The sections were then stained with Troponin T to mark cardiomyocytes before covered.

### BrdU administration

BrdU (Sigma, 19–160) was dissolved in 0.9% saline solution at a final concentration of 10 mg/ml as previously reported^48^ and administrated to the pregnant mice 7 times, once every two days before prior to the birthing period.

### FACS sorting

Adult (8–9 weeks old) and neonatal (P1-P3) mouse hearts were cut into small pieces (1–2 mm in diameter) and digested with 250 U/ml collagenase type 2 (Worthington, LS004176) and 0.3 U/ml Protease XIV (Sigma, P5147) to dissociate heart tissues for 30 min at 37 °C as previously reported^2^. Red blood lysis was performed using sterile filtered deionized water followed by two washing steps with PBS. Debris was removed with a 30 mm filter. The resulting single-cell suspension was incubated with blocking with 2% fetal calf serum in HBSS containing c-Kit (Abcam, ab25022) at 1:200 dilution cells for 30 min on ice and incubated for another 30 min with 2% fetal calf serum containing secondary antibody conjugated to Alexa flour 594. C-Kit^+^ cells were then sorted on a BD FACSAriaIII (BD Biosciences) with a flow rate of 500 cells/s, using a 130 μm nozzle by the configuration 594 nm laser.

### Activation of quiescent c-Kit^+^ cells

FACS-sorted c-Kit^+^ cells were cultured in CSC growth medium consisting of DMEM/F12 (Corning 10–092-CV) medium containing 10% FBS (Gibico), LIF (10 ng/ml, Millipore), insulin-transferrin-selenite (ITS, Invitrogen), bFGF (10 ng/ml, Peprotech), EGF (20 ng/ml, Peprotech), 1% pen-strep (Invitrogen), and 0.1% gentamicin (10 mg/ml liquid, Invitrogen). The CSC growth medium component was as previously reported^49^. After 48 h of culture, it was observed quiescent c-Kit^+^ cells had been activated, as demonstrated by proliferation activity.

### Overexpression of *Setd4*

On the basis of sequence of the mouse *Setd4* gene (NM_145482.3) in GenBank, a high titer (5 × 10^10^ PFU/ml) recombinant mouse adenovirus expressing Setd4-Green fluorescent protein under the control of CMV promoter (purchased from Vigenebiosciences) was efficiently transfected at 100 MOI in the activated c-Kit^+^ cells within 12 h, and then replaced with the fresh medium culturing for 48 h as previously reported^50^. A mouse adenovirus containing only GFP under the control of the CMV promoter was used for control experiments.

### Immunostaining

For heart section samples, hearts were collected and washed in cold PBS to remove excessive blood and fixed in 4% PFA at 4 °C. Hearts were then dehydrated in 30% sucrose in PBS at 4 °C until tissues were fully penetrated. Following one-hour immersion in optimum cutting temperature (O.C.T., Sakura) at 4 °C, the hearts were embedded in blocks and frozen at − 80 °C. Then, the embedded blocks were cut to a 10 μm thickness of each section. For cell samples, cells were fixed in 4% PFA at 4 °C and dropped on the slide after PBS washing. Samples were then blocked with PBS supplemented with 0.2% triton X-100 and 5% normal donkey serum for 30 min at room temperature, followed by primary antibody incubation at 4 °C overnight. The next day, samples were washed three times for 5 min each in PBS and incubated with secondary antibodies for 45 min at room temperature in the dark. After washing three times for 5 min each, samples were stained with DAPI as previously reported^15^. Images were taken using an Olympus confocal microscopy system (FV3000) or Zeiss confocal microscopy system (LSM710).

### Masson’s trichrome staining

Similar to previously described^51^, to determine infarct size, hearts were fixed in 4% paraformaldehyde (pH 8.0), dehydrated and embedded in optimum cutting temperature (O.C.T., Sakura) and frozen at − 80 °C. Then, the embedded blocks were cut through from apex to base. The first 10 sections (10 μm thickness each) of every 100 sections were used. Sections were further fixed with prewarmed Bouins’ solution at 55 °C for 1 h and stained with Masson trichrome stain. Infarct size was calculated according to the formula: [length of coronal infarct perimeter (epicardial + endocardial)/total LV coronal perimeter (epicardial + endocardial)] × 100.

### Antibodies

The following antibodies were used: Rat anti-BrdU (1:100, Abcam, ab6326); Rabbit anti-Ki67 (1:100, Abcam, ab16667); Rabbit anti-H3pS10 (1:150, Epitomics, 1173-1); Mouse anti-Setd4 (1:50, Santa Cruz Biotechnology, sc-514060); Rabbit anti-H4K20me3 (1:200, Abcam, ab9053); Mouse anti-HP1α (1:200, EMD Millipore Corp, 05-689); Rabbit anti-H3K9ac (1:200, Epitomics, 1328-1); Mouse anti-Troponin T (1:100, Invitrogen, MA5-12960); Mouse anti-sarcomerica-actin (1:100, Sigma, A2172); Rat anti-CD31 (1:200, BD Biosciences, 5553370); Goat anti-VE-cadherin (1:200, RD, AF1002); Rabbit anti-PDGFRα (1:100, RD, AF1062); Rabbit anti-Sox9 (1:100, Abcam, ab185230); Rabbit anti-α-SMA (1:150, Sigma, A2547); Rabbit anti-FABP4 (1:150, Abcam, ab92501); Rat anti-c-Kit (1:100, Abcam, ab25022).

### Western blots

The sorted cell samples were homogenized in RIPA buffer (50 mM Tris–HCl, 1 mM EDTA, 1 mM EGTA, 150 mM NaCl, 1% NP-40) containing protease inhibitor cocktail (Sigma-Aldrich, P8340). Ten micrograms of protein per sample were resolved on 10% SDS–polyacrylamide gel electrophoresis gels, transferred onto PVDF membranes, immunoblotted with antibodies for Setd4 (HuaAn Bio) at 1:100 dilution and Tubulin at 1:3000 dilution, and then incubated with the appropriate alkaline phosphate-linked secondary antibody as previously reported^52^. PVDF membranes were visualized using enhanced chemifluorescence (Amersham).

### Confocal microscope

Pictures were acquired using a confocal microscope (Leica, FV3000 or LSM710). Each picture was taken using the scan format of 1024 × 1024 pixels, speed at 400 Hz, image x and y dimensions of 238.10 mm, voxel-size of 231.51 × 3 nm, average line 1, average frame 3, and pinhole 1. The lasers considered were of 405 nm, 488 nm, 594 nm, and 647 nm. Each picture was adjusted to a width of 6 cm and a resolution of 300 pixels/inch.

### Quantification and statistical analysis

The studies were blinded during data collection and quantification. No data were excluded. All statistical analyses were performed in GraphPad Prism 8.0 software. For quantitative data analysis, two-tailed unpaired Student’s t test was used for two samples comparison (e.g., *Setd4*^+^ cells (%) in proliferating medium and CSC growth medium). One-way ANOVA was used to examine the effects of single factor among three groups (e.g., mEGFP^+^ cells (%) in E6.5 embryo, E15.5 heart and P14 heart). Two-way ANOVA was used to examine the effects of two categorical variables (e.g., TdTomato^+^ cells (%) in different regions of infarct heart after *Setd4* knock-out), followed by Bonferron’s post-test with multiple comparisons. All data are presented as mean ± SEM. The value of *p* < 0.05 was considered significant [*p* > 0.05 considered not significant (ns)].

### Ethics approval and consent to participate

All mice experiments were approved by and performed following the guidelines of the Institutional Animal Care and Use Committee at Zhejiang University.

## Supplementary Information

Supplementary Information.

## Data Availability

All datasets used and/or analyzed during this study are available from the corresponding author on reasonable request.

## Abbreviations

Setd4: Set domain-containing protein 4
H4K20me3: Trimethylation of lysine 20 of histone 4
H3K9ac: Acetylation of lysine 9 of histone 3
HP1α: Heterochromatin protein 1α
FACS: Florescence-activated cell sorting
ECs: Endothelial cells
MI: Myocardial infarction
EdU: 5-Ethynyl-2′-deoxyuridine
BrdU: 5-Bromo-2′deoxyuridine
FABP4: Fatty acid-binding protein 4
α-SMA: α-Smooth muscle actin
PI3K: Phosphatidylinositol-3-kinase
mTOR: Mammalian target of rapamycin
TUNEL: Terminal deoxynucleotidyl transferase-mediated dUTP nick-end labeling
