# Supplementary material for: Setd4 controlled quiescent c-Kit+ cells contribute to cardiac neovascularization of capillaries beyond activation
Source: Sci Rep. 2021 Jun 2;11:11603. doi: 10.1038/s41598-021-91105-6 (PMC8172824; doi:10.1038/s41598-021-91105-6)
Supplement: Supplementary file 1 — Supplementary Information. [file 41598_2021_91105_MOESM1_ESM.pdf]

Supplementary Information

Setd4 Controlled Quiescent c-Kit$^+$ Cells Contribute to Cardiac Neovascularization of Capillaries beyond Activation

Sheng Xing[1], Jin-Ze Tian[1], Shu-Hua Yang[1], Xue-Ting Huang[1], Yan-Fu Ding[1], Qian-Yun Lu[1], Jin-Shu Yang[1], Wei-Jun Yang[1]*

Affiliations:

[1]MOE Laboratory of Biosystem Homeostasis and Protection, College of Life, Sciences, Zhejiang University, Hangzhou, 310058, China.

***Corresponding author. Email: w_jyang@zju.edu.cn (W.Y.)**

Running title: Function and Regulation of Quiescent c-Kit$^+$ Cells

Supplementary Figure S1

Fig. S1

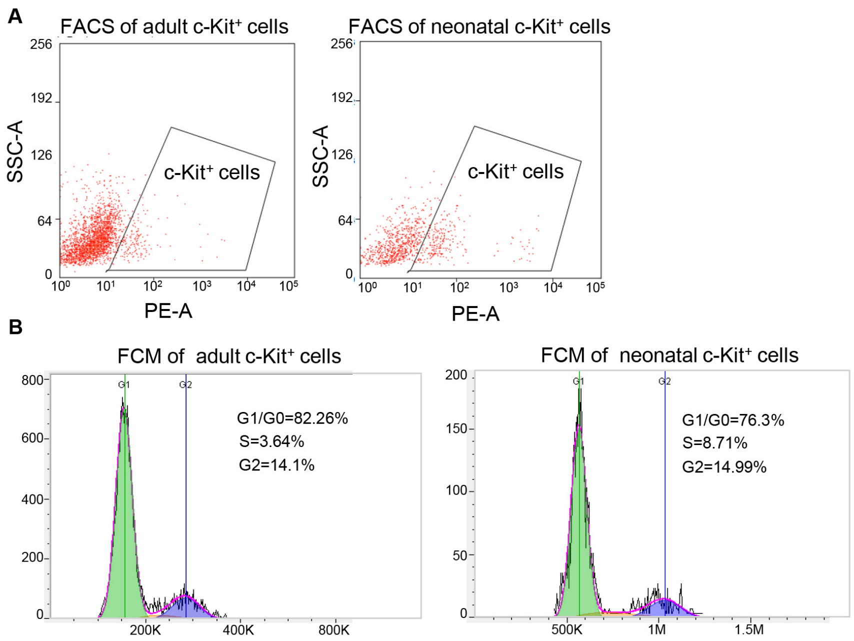

Figure. S1 Flow cytometry analysis of the c-Kit$^+$ cell population. **A,** Fluorescence-activated cell sorting (FACS) of c-Kit$^+$ cells from adult and neonatal hearts. **B**, Flow cytometry-based cell cycle analysis of c-Kit$^+$ cells of adult and neonatal hearts. All data are represented as mean ± SEM. n = 3 mice.

Supplementary Figure S2

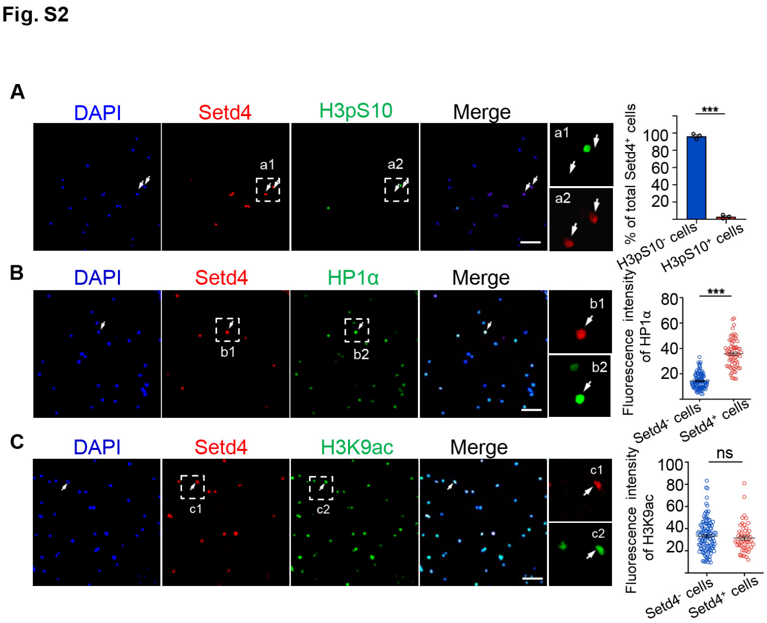

Figure. S2 Characterization of proliferation and chromatin in Setd4-expressing c-Kit^{+} cells. **A-C**, Representative immunofluorescence and quantification for c-Kit^{+} cells with Setd4 and cell proliferation marker, H3pS10 (**A**), n = 3 mice, heterochromatin marker, HP1α (**B**), n = 168 cells from 3 mice, and euchromatin marker, H3K9ac (**C**), n = 164 cells from 3 mice. Scale bars = 50 μm. Nuclei were stained with DAPI. All data are represented as mean ± SEM. Comparison of independent variables was conducted by two-tailed unpaired Student's t test. ***$p < 0.001$, ns: not significant.

Supplementary Figure S3

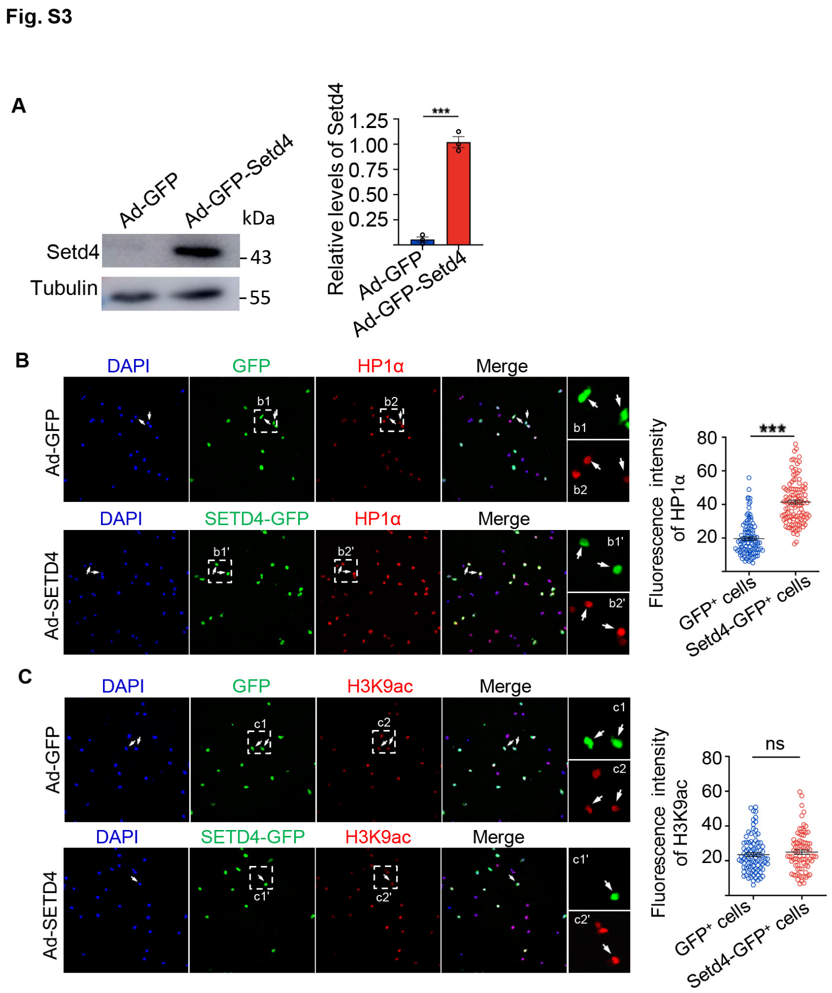

Figure. S3 Overexpression of *Setd4* in c-Kit^{+} cells facilitated heterochromatin formation. **A**, Western blot and quantification analysis of Setd4 expression after two days of *Setd4* overexpression. n = 3 mice. **B** and **C**, Representative immunofluorescence and quantification for activated c-Kit^{+} cells affected by Ad-GFP and Ad-Setd4 with HP1α (**B**), n = 220 cells from 3 mice, and H3K9ac (**C**), n = 169 cells from 3 mice. Scale bars = 50 μm. Nuclei were stained with DAPI. All data are represented as mean ± SEM. Comparison of independent variables was conducted by two-tailed unpaired Student's t test. ***$p < 0.001$, ns: not significant.

Supplementary Figure S4

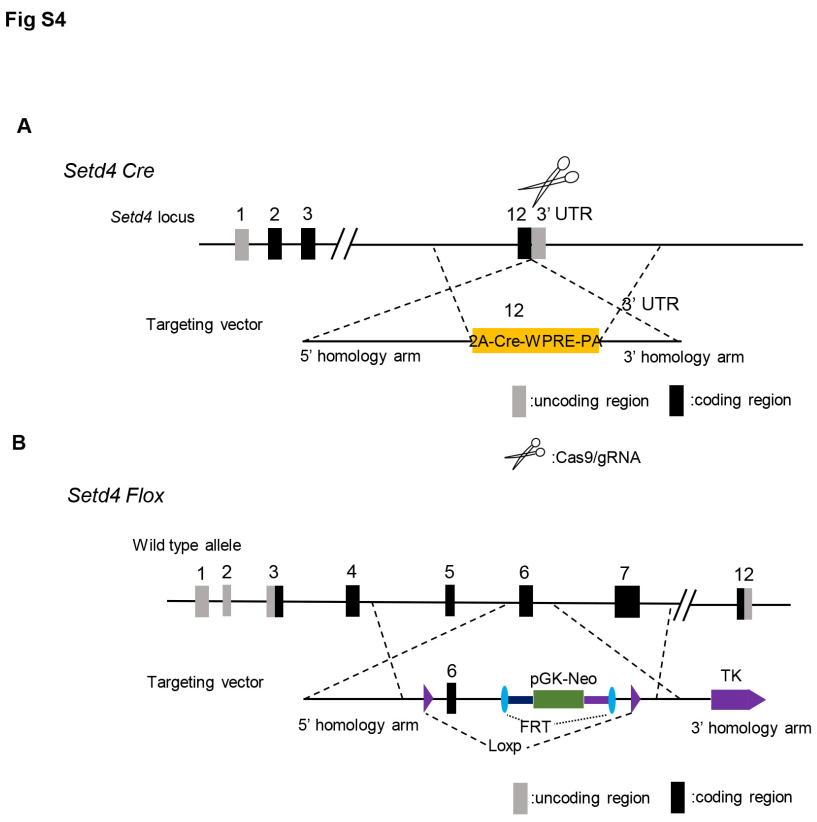

Figure. S4 Schematic diagram of design strategy of transgenic mouse line. **A**, Schematic diagram of design strategy of constitutive *Setd4-Cre* mouse line, generated by CRISPR/Cas9. The offspring of *Setd4-Cre* were mated with *Rosa26-TdTomato* mice. **B**, Schematic diagram of design strategy of conditional *Setd4* knock-out mouse line, with a pair of loxP sites flanking exon6 of *Setd4*. The offspring were mated with *c-Kit-CreER*^{T2} mice.

Supplementary Figure S5

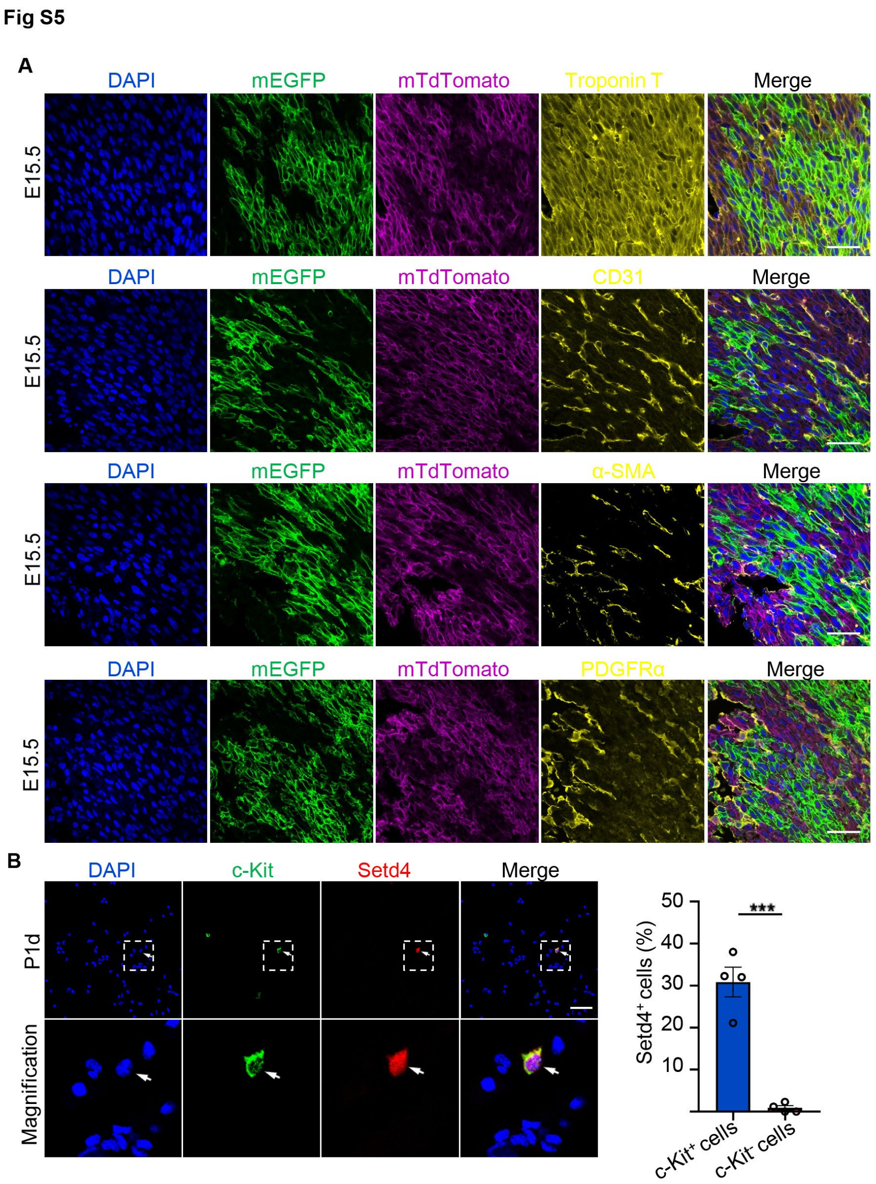

Figure. S5 Lineage tracing of Setd4^{+} cells in the E15.5 hearts of *Setd4-Cre;Rosa26*$^{mT/mG}$ mice. **A**, Representative immunofluorescence for recombinant mG^{+} cells with cardiomyocyte, EC, smooth muscle cell and fibroblast markers, Troponin T, CD31, α-SMA and PDGFRα, respectively, in the E15.5 hearts. **B**, Representative immunofluorescence and quantification for c-Kit^{+}Setd4^{+} cells of P1 heart cells. Scale bars = 50 μm. Nuclei were stained with DAPI. n = 4 mice.

Supplementary Figure S6

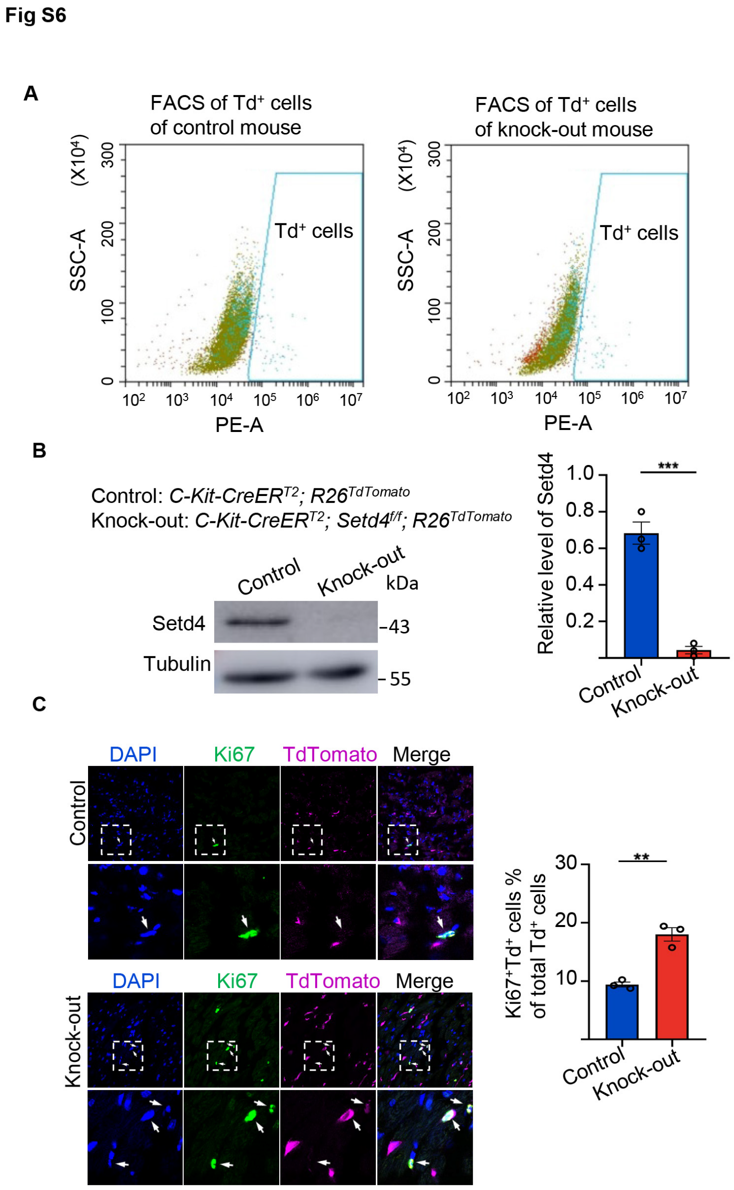

Figure. S6 Analysis for Setd4 knock-out efficiency and recombinant cells proliferation level after Setd4 knock-out. **A**, Fluorescence-activated cell sorting (FACS) of recombinant cells after 48-hour tamoxifen induction of adult hearts. **B**, Western blot and quantification analysis of Setd4 expression of sorted recombinant cells. **C**, Representative immunofluorescence and quantification for recombinant Td^+ cells with proliferation marker Ki67 in control and Setd4 knock-out hearts. All data are represented as mean ± SEM. n = 3 mice. Comparison of independent variables was conducted by two-tailed unpaired Student's t test. ***$p < 0.001$.

Supplementary Figure S7

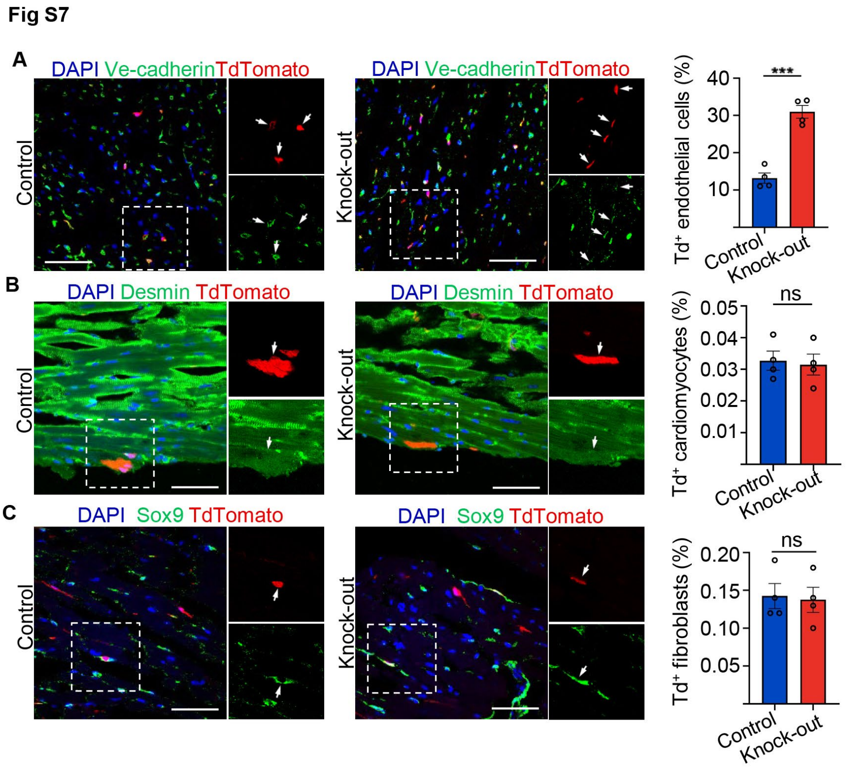

Figure. S7 Knock-out of Setd4 induced generation of newborn ECs in the adult mice. **A-C**, Representative immunofluorescence and quantification for recombinant cells with ECs, cardiomyocyte and fibroblast alternative markers, VE-cadherin (**A**), Desmin (**B**) and Sox9 (**C**). Scale bars = 50 µm. Nuclei were stained with DAPI. All data are represented as mean ± SEM. n = 4 mice. Comparison of independent variables was conducted by two-tailed unpaired Student's t test. ***$p < 0.001$, ns: not significant.

Supplementary Figure S8

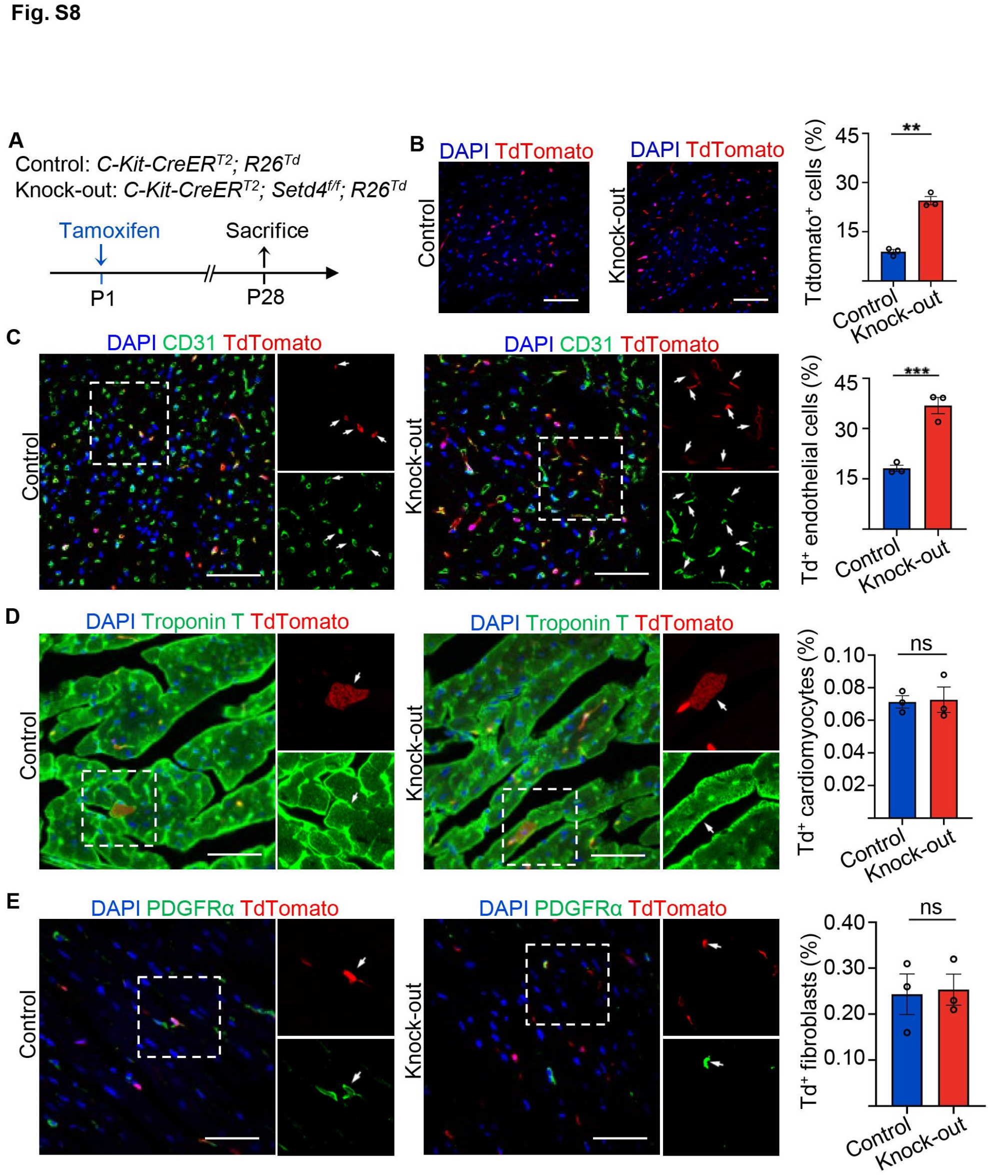

Figure. S8 Knock-out of *Setd4* induced generation of newborn ECs in the neonatal mice. **A**, Experimental outline for lineage tracing of neonatal mice. **B**, Representative immunofluorescence and quantification for recombinant cells after four weeks of *Setd4* knock-out. **C-E**, Identification and quantification for c-Kit cells-derived ECs (Td^+CD31^+) (**C**), cardiomyocytes (Td^+Troponin T^+) (**D**) and fibroblasts ($Td^+PDGFR\alpha^+$) (**E**) after four weeks of *Setd4* knock-out. Arrows indicate recombined cells and positive staining. Scale bars = 50 μm. Nuclei were stained with DAPI. All

data are represented as mean ± SEM. n = 3 mice. Comparison of independent variables was conducted by two-tailed unpaired Student's t test. **$p < 0.01$, ns: not significant.

Supplementary Figure S9

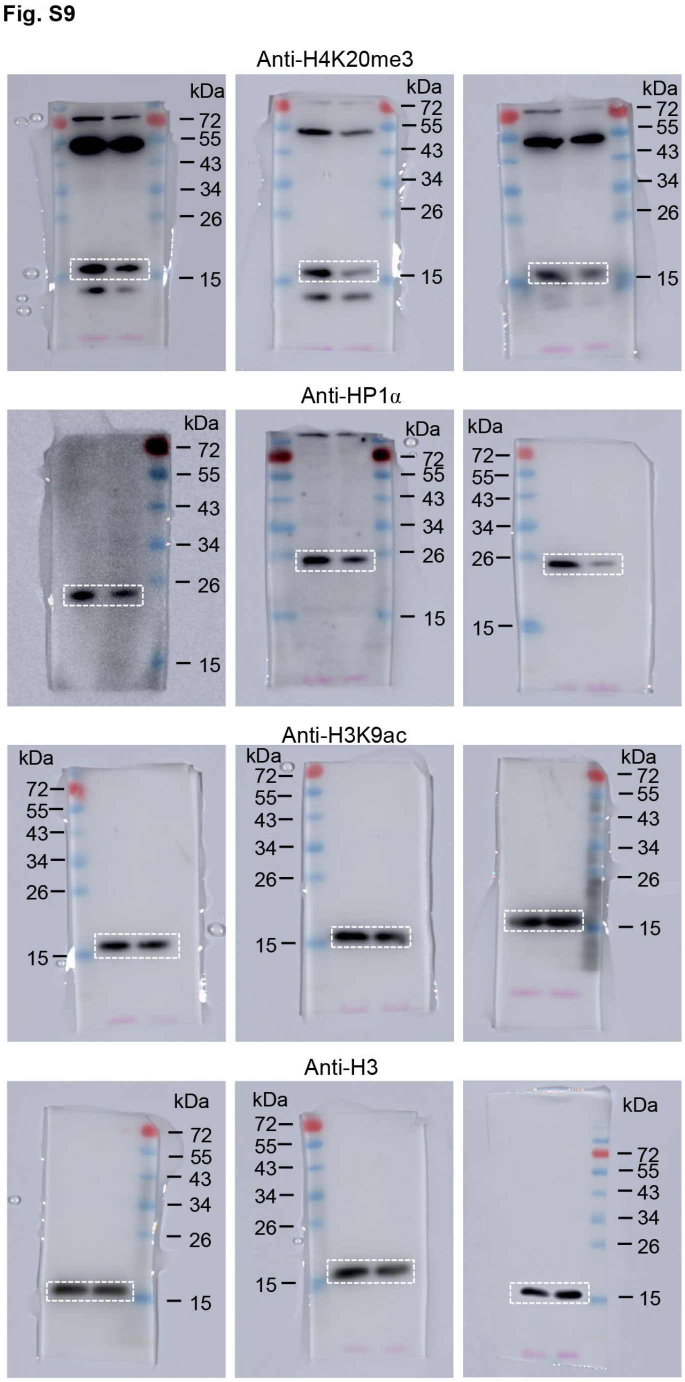

Figure. S9 Replicate uncropped gel scans for the presented western blots of histone modifications after Setd4 knock-out. Corresponds to Figure 5E.

Supplementary Figure S10

Fig. S10

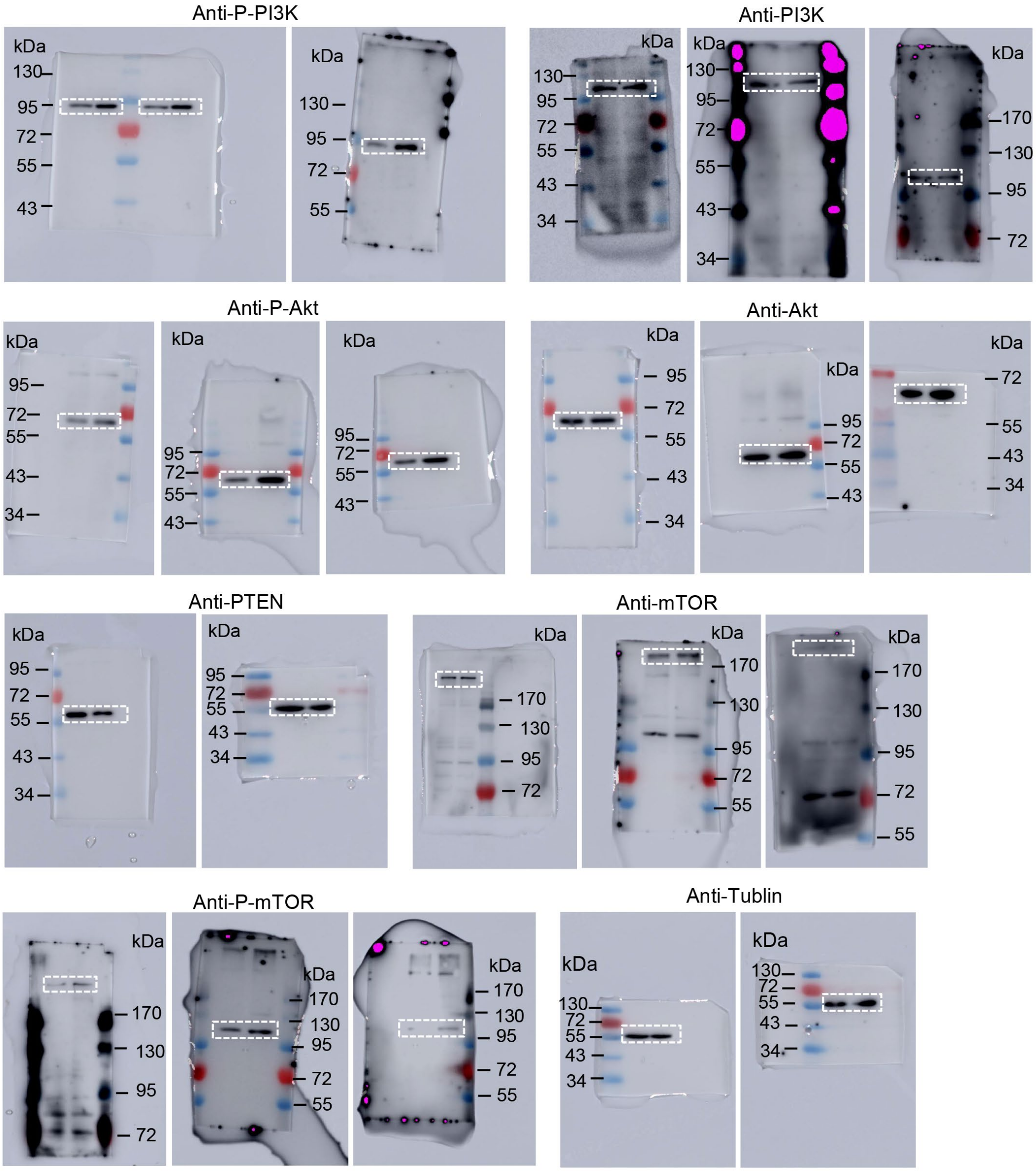

Figure. S10 Replicate uncropped gel scans for the presented western blots of the PI3K-Akt-mTOR signaling pathway after Setd4 knock-out. Corresponds to Figure 5G.

Supplementary Figure S11

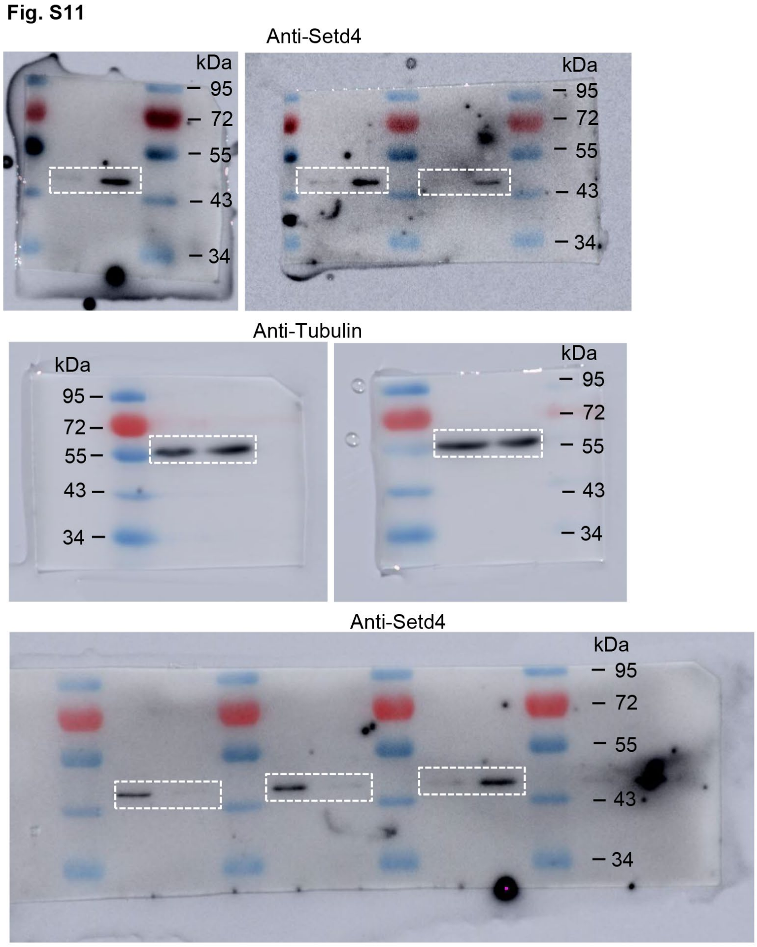

Figure. S11 Replicate uncropped gel scans for the presented western blots of Setd4 expression levels. Corresponds to Figure S3A and S6B.

Supplementary Figure S12

Fig. S12

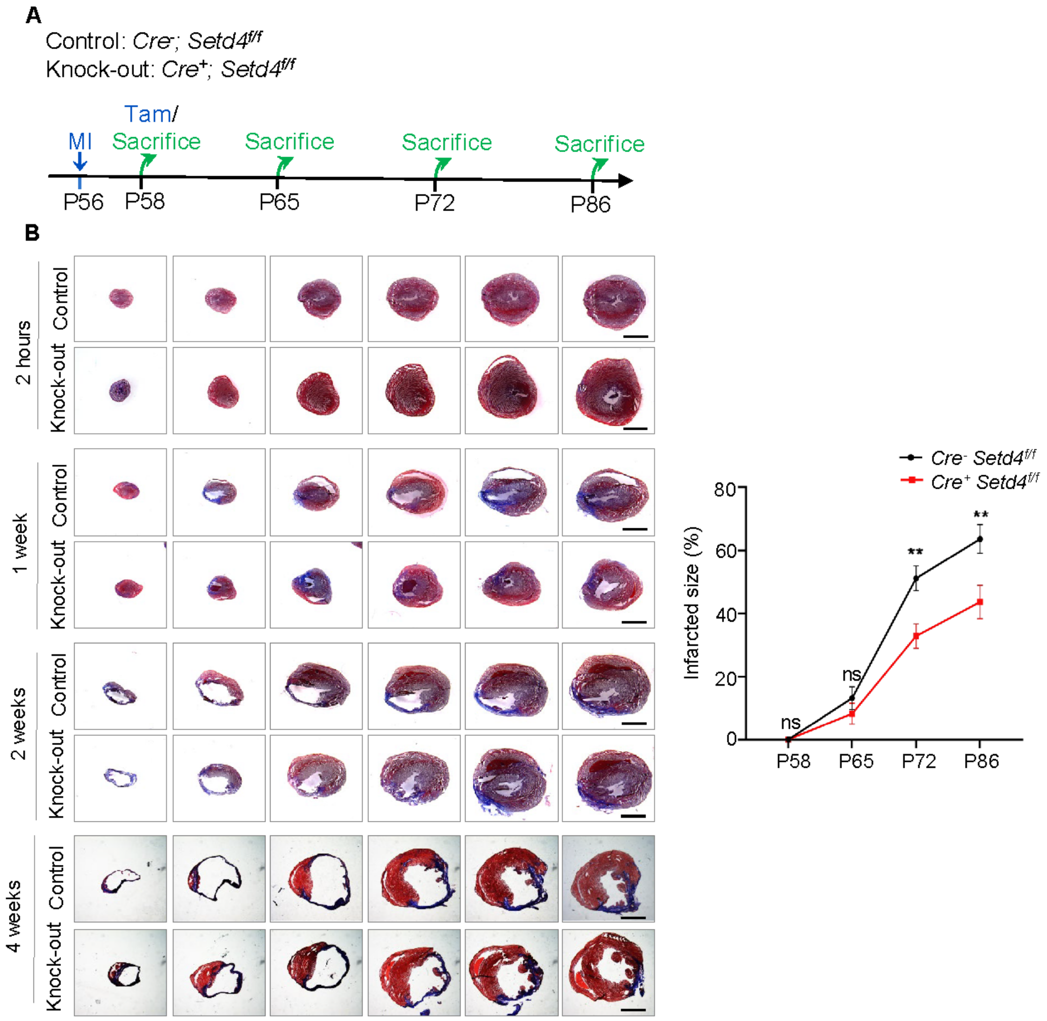

Figure. S12 Analysis of infarct size after *Setd4* knock out with increasing time. **A**, Experimental outline for injured hearts of *c-Kit-CreER*^{T2}*(Cre*$^{+}$*);Setd4*$^{f/f}$ and *c-Kit-CreER*$^{T2-}$*(Cre*$^{-}$*);Setd4*$^{f/f}$ mice. **B**, Masson trichrome staining and quantification for infarction size in hearts of *Setd4* knock-out and control mice after 2 hours, 1 week, 2 weeks and 4 weeks of Tamoxifen-induced. Scale bars = 2 mm. n = 4 mice. Error bars represent SEM. $^{**}p < 0.01$, ns: not significant; Multiple comparisons test was conducted by two-way ANOVA with Bonferroni's correction.
